# Greenland Emissions Contribute to Black Carbon Aerosols in the Arctic Marine Boundary Layer

**DOI:** 10.1021/acs.est.6c03343

**Published:** 2026-07-22

**Authors:** Xiaomi Teng, Mao Du, Congbo Song, Yuqing Dai, Vipul Lal Chandani, David C. S. Beddows, Manuel Dall’Osto, Darrel Baumgardner, Roy M. Harrison, James Brean, Mingxi Yang, Yangmei Zhang, Weijun Li, Zongbo Shi

**Affiliations:** † Department of Atmospheric Sciences, School of Earth Sciences, 12377 Zhejiang University, Hangzhou 310058, China; ‡ School of Geography Earth and Environmental Sciences, 1724 University of Birmingham, Birmingham B15 2TT, U.K.; § Department of Marine Biology and Oceanography, Institute of Marine Sciences (ICM, CSIC), Barcelona 08003, Spain; ∥ 232906 Droplet ENVEA Group, Longmont, Colorado 80503, United States; ⊥ 61564 Plymouth Marine Laboratory, Prospect Place West Hoe, Plymouth PL1 3DH, U.K.; # State Key Laboratory of Severe Weather/Key Laboratory of Atmospheric Chemistry of China Meteorological Administration, Chinese Academy of Meteorological Sciences, Beijing 100081, China; ○ National Centre of Atmospheric Science, School of Geography, Earth & Environmental Sciences, University of Birmingham, Edgbaston, Birmingham, B15 2TT, U.K.

**Keywords:** Arctic, black carbon, marine boundary layer, aerosols, anthropogenic emission

## Abstract

Black carbon (BC) is an important short-lived climate forcer that contributes to Arctic warming, yet observations of BC in the Arctic marine boundary layer remain sparse. Here, we report shipborne measurements of refractory BC (rBC) mass and particle mixing state during a cruise from southeast to west Greenland in May–June 2022. Using a single-particle soot photometer (SP2XR) and transmission electron microscopy (TEM), we characterize both rBC concentrations and individual-particle morphology, representing one of the first ship-based SP2XR data sets in the Arctic marine boundary layer. rBC concentrations were highest in the Greenland coastal region (2.4 ng m^–3^), compared with the open ocean (1.7 ng m^–3^) and the marginal ice zone (0.6 ng m^–3^). Mixing-state analysis shows that 54% of soot particles in coastal air masses were externally mixed, consistent with relatively fresh emissions, whereas most soot particles over the open sea and sea-ice regions were internally mixed, indicative of aged, transported aerosol. FLEXPART modeling suggests BC enhancements along the Greenland coast. Our data indicate that Greenland acts as a regional source of BC to the Arctic marine boundary layer in late spring and early summer, when midlatitude transport is weak.

## Graphical abstract

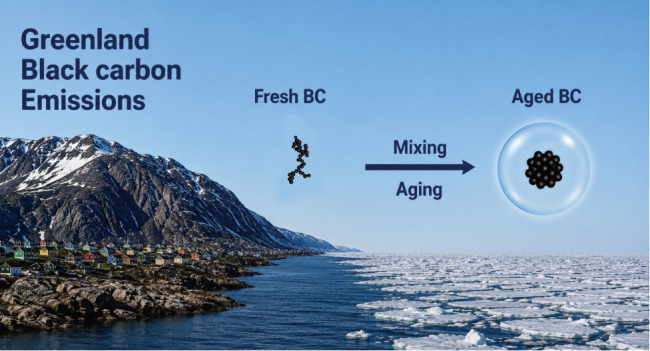

## 1 Introduction

The Arctic is highly sensitive to climate change, warming nearly four times faster than the global average.^1^ This accelerated warming has implications for the global climate system, as it can alter atmospheric dynamics and contribute to extreme weather events in midlatitude regions.^2^ Black carbon (BC), as a significant short-lived climate forcer, can absorb sunlight, whether suspended in the atmosphere or deposited on snow and ice surfaces.^3^ This absorption results in increased air temperatures, earlier ice melt during the spring, and an extended melting season.^4,5^ However, uncertainties remain about the BC properties in the Arctic, particularly the baseline and emission sources in the marine boundary layer. Increased shipping in the Arctic is expected to elevate black carbon emissions, leading to greater deposition of BC on snow and ice.^6,7^

Current BC observations are mostly made from ground-based monitoring sites, with occasional aircraft and ship campaigns.^8,9^ However, in situ measurement sites on land cannot fully represent regional BC properties across the Arctic, where nearly 70% of the area is covered by ocean or ice.^10^ Aircraft campaigns are effective for obtaining vertical profiles but have limited ability to capture near-surface BC properties.^3^ Satellite data provide information on aerosol properties near the sea surface but are limited by coarse spatial resolution.^11^ Therefore, shipborne BC measurements are valuable to capture the properties of BC near the sea surface and enhance our understanding of its near-surface distribution. Although shipborne BC observations have been conducted in regions such as the Western Arctic Ocean,^12–14^ the Bering Sea,^14^ Svalbard,^15^ and along the Russian Arctic coast,^16,17^ observations near Greenland remain scarce. Therefore, further shipborne BC measurements near Greenland are critical for accurately assessing the impact of Arctic aerosols on climate.

There are large uncertainties regarding BC emission sources in the Arctic, which contribute to the disagreement between modeled and observed BC.^18,19^ Aerosols in the Arctic originate mainly from long-range transported anthropogenic sources during winter and from local natural sources during summer.^20^ BC in the Arctic marine boundary layer originates mainly from fossil fuel sources in Russia and Europe during winter and from biomass burning (e.g., wildfires) in Siberia, Alaska, and Canada during summer.^21–23^ Previous studies generally showed a limited role of the local anthropogenic sources in the Arctic because of low-intensity human activities. However, some studies suggest that inner-Arctic anthropogenic emissions have a more pronounced impact on the Arctic climate than long-range transported emissions.^24,25^ The locally emitted BC is more readily deposited on the ice surface, increasing surface temperature and altering the vertical profile of the Arctic atmosphere, while transported BC rarely penetrates the low troposphere through the polar dome.^20,26,27^ Therefore, understanding local pollutant emissions is crucial for assessing aerosol-climate interactions in the Arctic.

In this study, we carried out a shipborne observation campaign to investigate BC properties in the marine boundary layer to the southeast and west of Greenland. We employed an extended-range single-particle soot photometer (SP2XR) and an Aethalometer to determine the time series of refractory black carbon (rBC) and equivalent black carbon (eBC) mass concentrations, respectively. Transmission electron microscopy (TEM) was used to analyze the microscopic physicochemical properties of individual BC particles. The primary objective of this study was to characterize BC particles from coastal regions to the open sea and the marginal ice zone.

## 2 Methods

### 2.1 Shipboard Aerosol Campaign

The data were collected aboard the *Royal Research Ship (RRS) Discovery* from 20th May to 23rd June 2022. The cruise route started from Reykjavik, Iceland, and traversed the North Atlantic Ocean, traveling to nearby cities such as Nuuk, Maniitsoq, and Sisimiut before reaching the ice edge at the southern tip of Baffin Bay and returning to Southampton, UK (Figure 1a and S1). The mass concentrations of fine particles (PM_2.5_), refractory black carbon (rBC), and equivalent black carbon (eBC) were measured using a Fidas (Model Fidas 200, PALAS, Germany), a single-particle soot photometer (SP2XR, DMT, Boulder, CO, USA), and an Aethalometer (Model AE33, Magee Scientific, USA) at 880 nm. Nafion dryers were installed upstream of the AE33 and SP2XR, respectively, to reduce sample relative humidity and minimize potential RH-related biases in the measurements. AE33 was equipped with a PM_2.5_ size-selective inlet operating at a flow rate of 5 L min^–1^. The AE33 Aethalometer data were corrected for multiple scattering using a correction factor C = 2.95, determined dynamically from the filter loading parameter k following Ferrero et al. (2024).^28^ A field-calibrated mass absorption cross-section MAC = 8.8 m^2^ g^–1^ at 880 nm was adopted based on Arctic aerosol measurements.^29^ During the cruise, all online instruments were mounted in air-conditioned (18 °C) containers positioned on the foredeck of the ship, approximately 18 m above sea level. The ship’s exhaust funnel was behind the bridge and higher up on the ship. More details of the campaigns are shown by Du et al.^30^

**Figure 1:**
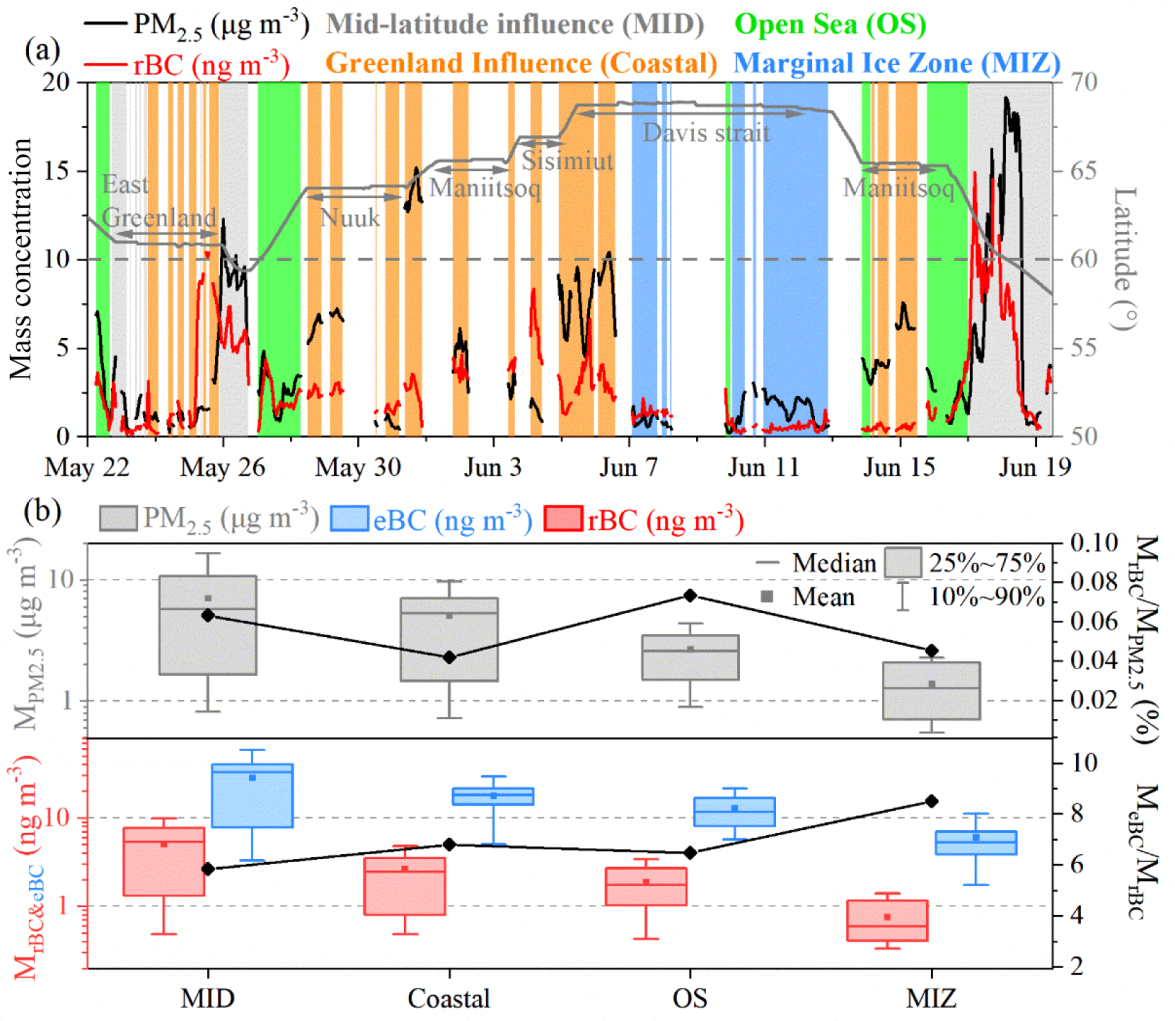
Spatial and temporal distribution of PM_2.5_ and rBC mass concentrations during the Arctic cruise. (a) The time series of PM_2.5_ and rBC mass concentrations. Blank segments indicate data affected by the ship’s own emissions and were excluded. Colored shades indicate the four regions. (b) PM_2.5_, eBC, and rBC mass concentrations and the relative contribution of rBC to PM_2.5_ in four Arctic regions: the midlatitude-influenced region (MID), the Greenland Coastal region (Coastal), open sea (OS), and Marginal Ice Zone (MIZ). The four regions are indicated by shaded areas in the time series (a) and by dashed boxes in (b). Box plots represent the interquartile range (25%–75%) and 10%–90% range, with horizontal lines showing medians and squares indicating means. The black dots and connecting line represent the mass concentration of rBC relative to PM_2.5_ (M_rBC_/M_PM2.5_, %) and the relative mass concentration of eBC to rBC (M_eBC_/M_rBC_).

To minimize the influence of ship exhaust, we masked periods when ship-stack emissions were being sampled based on meteorological data, mixing ratios of nitrogen oxides (NO_*x*_) and sulfur dioxide (SO_2_), and rBC. The relative wind speed (RWS) and relative wind direction (RWD) were defined with respect to the ship’s heading. RWS represents the wind speed measured at the sampling inlet relative to the vessel, while RWD is the wind direction relative to the bow, with 0° indicating wind coming from the bow and 180° from the stern. The RWS and RWD were used to determine when the ship emissions were likely to affect the sampling inlets (RWD between 157.5° and 202.5° or RWS below 3 m s^–1^). After data screening, 61% of the original data was retained.

### 2.2 Individual Sample Collection and TEM Analysis

Individual aerosol samples were collected onto copper TEM grids coated with carbon film (carbon type-B, 400-mesh copper; Tianld Co.) using a double-stage cascade impactor (DKL-2, Genstar Electronic Technology, China) with 0.5 mm and 0.3 mm diameter jet nozzles at 0.8 L min^–1^. The upper stage (0.5 mm nozzle) mainly collects coarse aerosols, while the lower stage (0.3 mm nozzle) captures finer, submicron particles, effectively separating supermicron from submicron aerosols. The fine-mode stage has a 50% collection efficiency at 0.08 μm, and the coarse-mode stage has a 50% collection efficiency at 0.3 μm, assuming a particle density of 2 g cm^–3^ by the air flow rate of 1 L min^–1^.^31^ This study focused on the second-stage samples (0.3 mm) for fine particle analysis, as preliminary results indicated that the first-stage samples primarily contained coarse sea salt particles (Figure S2). Sampling durations varied from 60 to 90 min depending on the meteorological conditions and particle concentrations. To minimize the influence of emissions from the RRS Discovery, offline sample collection was restricted to periods identified as clean based on the same screening criteria described above. After collection, the samples were stored in dry (approximately 20% RH), clean, and airtight containers for subsequent laboratory analysis.

Individual aerosol particles were analyzed using transmission electron microscopy (JEOL JEM-2100, Japan) operated at 200 kV and equipped with energy-dispersive X-ray spectrometry (TEM/EDX). This technique allows for the examination of particle morphology, chemical composition, and the mixing state of individual particles. The equivalent circle diameters of individual particles were measured by using the image analysis software Radius (EMSIS GmbH, Germany). In total, 3337 aerosol particles in 11 selected individual particle samples (Table S1) were analyzed. Detailed classification criteria are provided in Text S1 and Figure S3 of the Supporting Information and by Li et al. (2011).^32^ TEM can easily identify BC particles due to their unique morphology of grape-like aggregates (Figures S4a and S5). Here, we use BC to denote mass concentrations derived from bulk measurements, while soot refers to BC particles identified by the TEM analysis.

### 2.3 Back Trajectories

To identify potential sources and transport pathways of Arctic aerosols, we used the Lagrangian particle dispersion model FLEXPART (version 8.2).^33^ This model computes backward particle footprints based on atmospheric turbulence, convection, particle deposition, and reactions with hydroxyl radicals.^33^ The model was run using 6-hourly meteorological data with a 0.5° × 0.5° resolution and 50 vertical levels, derived from the NCEP (https://rda.ucar.edu/datasets/ds094.0/, last access: 19th August 2023). Simulations were run 10 days backward, which covers the typical BC lifetime (∼1 week).^3^ The simulations were calculated every 6 h (00:00, 06:00, 12:00, 18:00 UTC) at an altitude of 10 m above sea level.

Additionally, 72-h back trajectories were calculated using the NOAA Hybrid Single-Particle Lagrangian Integrated Trajectory Model (HYSPLIT).^34^ Meteorological input data were obtained from the Global Data Assimilation System (GDAS) 1-degree archive (Dec 2004—present) provided by the NOAA Air Resources Laboratory (ARL). Trajectories were calculated starting at 10 m a.g.l.

### 2.4 Classification of Sampled Aerosols

In order to investigate the spatial variations of aerosols, we divided observations into four main categories based on sampling locations and air mass back trajectories (Figure S6a). This classification facilitated the analysis of aerosol properties under different environmental conditions in the Arctic.

#### 2.4.1 Midlatitude Influenced (MID)

The ship was located south of 60°N, or the air mass spent more than 30% of the previous 72 h south of 60°N.

#### 2.4.2 Greenland Coastal Region (Coastal)

The air mass spent time over the west coast of Greenland during the last 72 h, or the distance from the west coast below 200 km.

#### 2.4.3 Open Sea (OS)

The air mass spent most of the time over the open sea surface during the last 72 h. Trajectories passing over the northern Canadian Arctic islands are also classified into this category as these regions are largely uninhabited and have minimal anthropogenic influence.

#### 2.4.4 Marginal Ice Zone (MIZ)

The region between the open ocean and inner pack ice,^35^ with criteria of sea ice concentration between 10%–80%^36^ (Figures S7-S8). The air mass spent most of the time over the ice surface and came from the northern Arctic regions, distant from land and urban areas.

## 3 Results

### 3.1 Mass Concentration of PM_2.5_ and rBC

Figure 1a presents the time series of PM_2.5_ and rBC mass concentrations during the cruise. PM_2.5_ mass concentrations ranged from 0.17 to 19.1 μg m^–3^, while rBC mass concentrations ranged from 0.11 to 14.9 ng m^–3^ (Figure 1a). Figure S1a–b shows the geographical distribution of PM_2.5_ and rBC mass concentrations from the southeast to the west of Greenland during the cruise, respectively. Mass concentrations of PM_2.5_ and rBC were higher at lower latitudes (<60°N) and decreased with increasing latitude (Figure 1a). To analyze aerosol properties in the Arctic marine boundary layer, we defined the Greenland Arctic as the region where the latitude was above 60°N and the air mass back trajectories remained above 60°N during the previous 72 h.

The median PM_2.5_ mass concentration in the MIZ was 1.3 μg m^–3^ with a standard deviation of 0.7, likely representing the background levels in the Greenland Arctic during early summer (Figure 1b and Table S2). The median PM_2.5_ mass concentration in open sea (OS) air was 2.6 μg m^–3^ with a standard deviation of 1.5 (Figure 1b and Table S2). In the Greenland coastal region (Coastal) air, the median PM_2.5_ mass concentration was even higher at 5.3 μg m^–3^ with a standard deviation of 3.7, which is 4 times that for MIZ and 2 times that for OS air (Figure 1b and Table S2). The PM_2.5_ mass concentration in midlatitude-influenced air masses had a median of 5.7 μg m^–3^ with a standard deviation of 5.8 (Figure 1b and Table S2), slightly higher than in Coastal air masses. In contrast, the rBC mass concentration decreased markedly from 5.3 ng m^–3^ in midlatitude-influenced air to 2.4 ng m^–3^ in Coastal air masses, corresponding to a 55% reduction. The overall patterns of variations in observed rBC mass concentrations within the Arctic were similar to those of PM_2.5_ (Figure 1b). The median rBC mass concentration in the coastal region was 4 times that of MIZ (0.6 ng m^–3^) and 1.4 times that over open sea (1.7 ng m^–3^). These results indicate a clear decreasing trend in aerosol loading from the coastal regions through the open sea to the marginal ice zone in the Greenland Arctic.

Figure 1b also shows that the mass fraction of rBC relative to PM_2.5_ of MID, Coastal, OS, and MIZ aerosols shows consistent values of 0.06%, 0.04%, 0.07%, and 0.05%, respectively. These results show that rBC constitutes a very small fraction (<0.1%) of PM_2.5_ mass in the Arctic marine boundary layer.

During this campaign, we simultaneously operated an Aethalometer to obtain the eBC mass concentrations. The median eBC mass concentration was 18.2 ng m^–3^ in the coastal region, 11.6 ng m^–3^ over the open sea, and 5.3 ng m^–3^ in the MIZ (Figure 1b and Table S2), showing a decreasing gradient from coastal to MIZ. The eBC in MID air is even larger at 32.4 ng m^–3^. Here, we observed distinct differences between rBC and eBC mass concentrations in the Arctic region. The time series shows that the eBC mass concentrations are consistently higher than the rBC mass concentrations (Figure S9). Linear regression between eBC and rBC yielded a slope of 3.8 (R^2^ = 0.56) with a positive intercept of 6.85 ng m^–3^ (Figure S10). The slope is consistent with the range of 1.5–5 reported in previous Arctic studies.^7,37^ The overall median eBC/rBC ratio was 8.8, while the regional median ratios were 5.8, 6.8, 6.5, and 8.5 for midlatitude-influenced, Coastal, Open Sea, and MIZ aerosols, respectively (Figure 1b and Table S2). The results show that the difference between eBC and rBC is more distinct in the MIZ, indicating that the cleaner the environment, the greater the difference between eBC and rBC mass concentrations.

### 3.2 Mixing States of Individual BC Particles

On the basis of the morphology, mixing state, and chemical composition of individual particles collected during the cruise, we classified them into five major types: soot-containing (Figure 2a,b), mineral (Figure S4c-d), sea salts (Figure S4e-m), sulfur-rich (S-rich) (Figure S4n-o), and other particles (Figure S4p-s). In this study, we found that soot-containing, sea salt, and S-rich particles were the dominant aerosol types by number (Figure S11a). Soot-containing particles accounted for 37% of the aerosol number concentration in the coastal region, 22% over the open sea, and only 7% in the MIZ. The differences in the number fraction of soot-containing particles are qualitatively similar to variations in the bulk rBC mass concentration.

**Figure 2:**
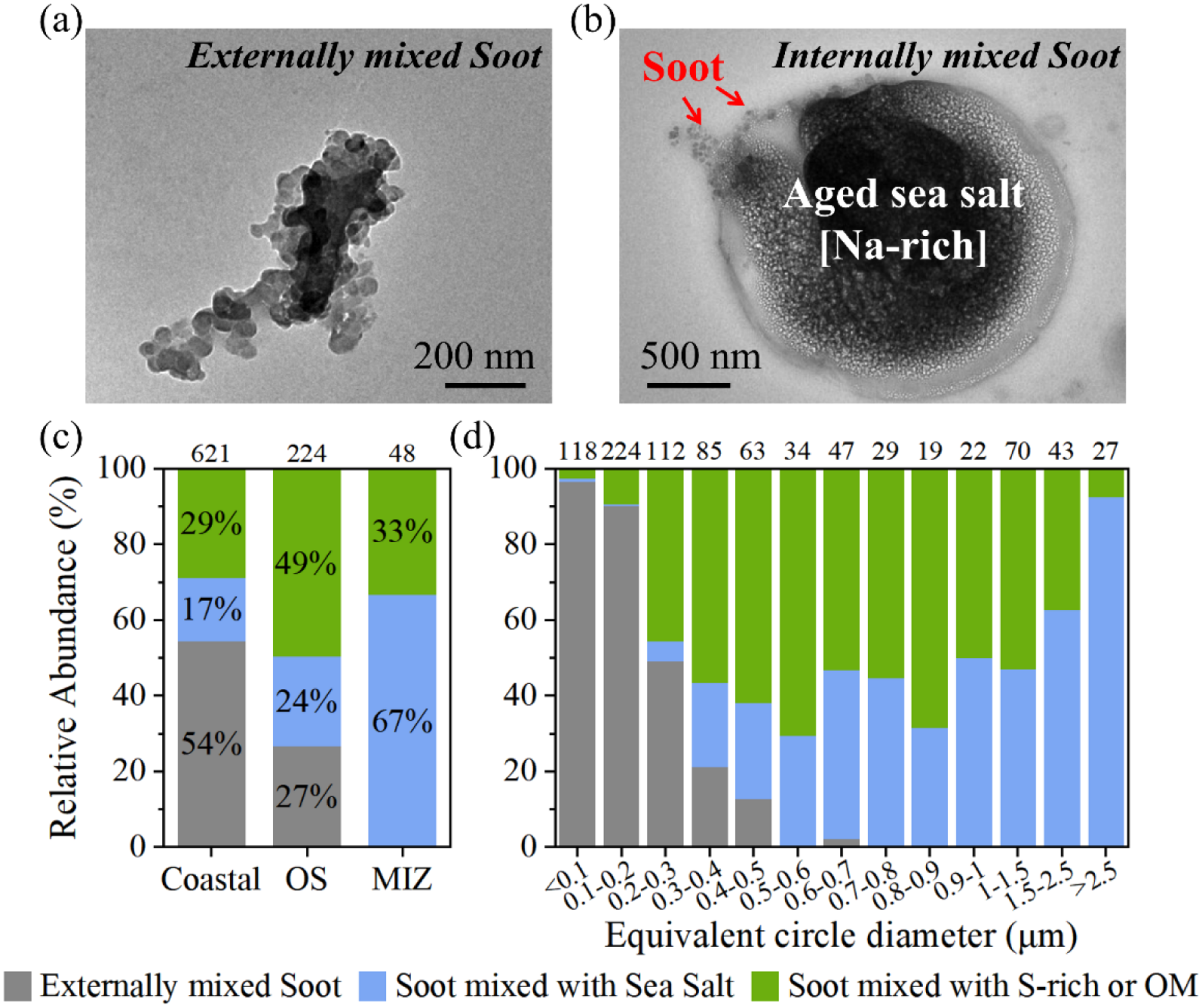
TEM characterization of major particle types and their size distributions. Representative TEM images of soot-containing particles: (a) externally mixed soot and (b) internally mixed soot. (c) The number fraction and (d) the size distribution of soot-containing particles in the three regions. The number of analyzed particles is shown above each column.

We observed both externally mixed (Figure 2a) and internally mixed BC (Figure 2b) in the Arctic marine boundary layer. Figure 2 shows that 54% of BC particles in coastal regions were externally mixed, compared to 27% over the open sea and none in the MIZ. All soot-containing particles in the MIZ and the majority (73%) of those over the open sea were internally mixed with sea salt (Figure 2b) and sulfate (S-rich) (Figure S4n), respectively (Figure 2c). Notably, BC particles internally mixed with aged sea salts (e.g., NaNO_3_ and Na_2_SO_4_) were most abundant in the MIZ, accounting for 67% of total soot-containing particles, followed by 24% over the open sea and 17% in coastal regions (Figure 2c). These results show a spatial variation of BC mixing state: BC is predominantly externally mixed in coastal regions and internally mixed over the sea surface (Figure 2c).

Soot-containing particles show bimodal size distributions (Figure S12). The smaller-size mode peaks at 141 nm and represents externally mixed BC, while the larger-size mode peaks at 648 nm and corresponds to internally mixed BC. Soot-containing particles were enriched in both small and large sizes, accounting for 75% by number in the <0.1 μm size bin and 40%–50% in the >1 μm size bins (Figure S11b). Figure 2d shows that soot-containing particles at small sizes (<0.1 μm) are primarily externally mixed, accounting for 97% by number, while those at larger sizes (>1 μm) are mainly internally mixed with coarse sea salt particles, accounting for 93% by number. This result agrees with previous findings that internally mixed BC particles are larger than externally mixed ones because coating materials increase their size.^38^ In the coastal region, BC particles are predominantly externally mixed, with smaller particle sizes and a peak around 141 nm (Figure S13a). In contrast, BC in the open sea and MIZ is mainly internally mixed, leading to larger particle sizes with peaks ranging from 346 to 548 nm (Figure S13b). The differences in BC particle size further highlight the spatial variation of BC in the Arctic.

## 4 Discussions

### 4.1 BC Observations in the Arctic

Large differences between the eBC and rBC mass concentrations were observed in the Arctic (Figure 1 and S9-S10). eBC mass concentrations are much higher than rBC in the background MIZ (Figures 1b). Our observed rBC mass concentrations over the open sea and in the MIZ during early summer ranged from 0.6 to 1.7 ng m^–3^, which is comparable to shipborne measurements reported over the western Arctic Ocean (70–75° N, ∼170–156° W) during September.^13,14^ In filter-based eBC measurements, non-BC aerosols such as dust and sea salt can enhance light scattering,^3^ while light-absorbing organic aerosols (e.g., brown carbon) can contribute to absorption;^39^ both can lead to increased apparent absorption that results in overestimated eBC mass concentrations. In this study, the median absorption Ångström exponent (AAE_470/880_ = 0.915) is close to unity (Figure S15), and few tar ball particles were identified in TEM analysis (Figure S16), suggesting negligible contributions from brown carbon or tar ball–type organic aerosols. However, submicron dust and sea salt particles were frequently observed (Figure S11a). Under such low-BC conditions, the relative contribution of non-BC scattering aerosols becomes more important, which probably led to a systematic overestimation of eBC.

Aethalometer-derived eBC is based on assumed MAC and multiple-scattering correction factors, both of which vary with aerosol type and environmental conditions.^7^ Multiple scattering in filter-based instruments occurs when the incident light is scattered by the filter fibers, resulting in more frequent interceptions of light by the embedded absorbing particles.^40^ Nonabsorbing aerosols can further increase multiple-scattering correction factors in aged air masses.^40^ Following Ferrero et al. (2024),^28^ dynamic multiple-scattering correction factors derived from our data set ranged from 2.13 to 4.65 and increased toward higher latitudes (Figure S17b). A campaign-mean filter-loading parameter was adopted to derive campaign-mean multiple-scattering correction factor of 2.95 for the entire campaign, which agrees well with previously reported Arctic values that increased from 2.40 near Tromsø to 4.28 at Ny-Ålesund and 5.26 over the open Arctic Ocean.^28^ However, persistently negative filter-loading parameter values at 470 nm during 9–23 June prevented reliable calculation of dynamic multiple-scattering correction factors. This period corresponded to the highest single-scattering albedo conditions during the campaign, suggesting that the multiple-scattering correction factor may be underestimated in the remote MIZ region.

The MAC value of 8.8 m^2^ g^–1^ at 880 nm was adopted from Ohata et al. (2021),^29^ which was derived from Arctic land-based observations closer to emission sources. Aerosol aging and internal mixing during transport can enhance BC absorption through the lensing effect, potentially increasing MAC in remote Arctic air masses.^6^ The increasing eBC/rBC ratio from the coast to remote MIZ supports this interpretation. This suggests that the MAC value we have adopted from Ohata et al. (2021)^29^ may be an underestimate of the true MAC for our ship observations.

Therefore, the higher eBC than rBC is likely caused by underestimated multiple-scattering correction factors and MAC values, resulting from strong aerosol scattering and the lensing effect in the Arctic marine boundary layer. The general overestimation of eBC relative to that of rBC, particularly in pristine environments, highlights the limitations of optical measurements at low BC concentrations.

In contrast, SP2 detects rBC through laser-induced incandescence and is less affected by non-BC components. Although SP2 may underestimate BC mass concentration because of size detection limits (50–800 nm for SP2XR in this study), this bias is better constrained since most BC particles are within this size range.^41^ Potential positive bias in SP2 measurements resulting from laser-induced charring of organic aerosols has been reported,^42^ but such effects are expected to be minimal in this study based on the low AAE values (Figure S15) and TEM observations (Figure S16). As a result, the SP2 measurement of BC in the atmosphere has a lower uncertainty of 10%–25% than the Aethalometer measurement uncertainty of 50%–80%.^3^ However, most studies relied on optical methods to determine BC mass concentration in the Arctic.^7,43–48^ The eBC mass concentration tends to significantly overestimate the BC mass in the pristine Arctic, leading to an overestimation of the BC baseline. The observational challenges in measuring BC at low concentrations cause significant uncertainties in BC observations in the polar regions.

Apart from instrumental biases, significant uncertainties in background BC also stem from the limited representativeness of land-based observations for the entire Arctic region. Current observations in the Arctic mainly originated from land-based stations, with sparse shipborne data available around Greenland (Figure S18). Our shipborne observation captured a background rBC mass concentration of 0.6 ng m^–3^ over the sea surface in the MIZ during early summer (Figure 1b), providing data for background conditions over the open ocean. Meanwhile, although we observed a high rBC mass concentration of 2.4 ng m^–3^ in coastal aerosols (Figure 1b), the values remain lower by one order of magnitude than rBC mass concentrations of 20 ng m^–3^ observed at Station Nord in northern Greenland in May.^49^ Repeated spikes in summer BC time series at land-based sites have been reported in previous studies, with no clear explanation provided.^7,37,45,49^ Local emissions could partly explain these peaks, which may result in an overestimation of background BC mass concentrations in the Arctic. Therefore, our results emphasize the need for SP2 or SP2XR measurements and for shipborne observations to better characterize background BC in the Arctic.

### 4.2 Greenland as a Source of BC

The FLEXPART back trajectories show that the air masses along the cruise track primarily originated from the west coast of Greenland and the open sea between northern Canada and Greenland (Figure 3). This suggests that BC in the Greenland Arctic region during early summer was mainly influenced by sources within the Arctic, with limited contributions from lower midlatitudes. These results support the established view that air pollution from midlatitudes was limited by the Arctic dome during summer, resulting in a higher local emission contribution to the surface BC.^3,50^

**Figure 3:**
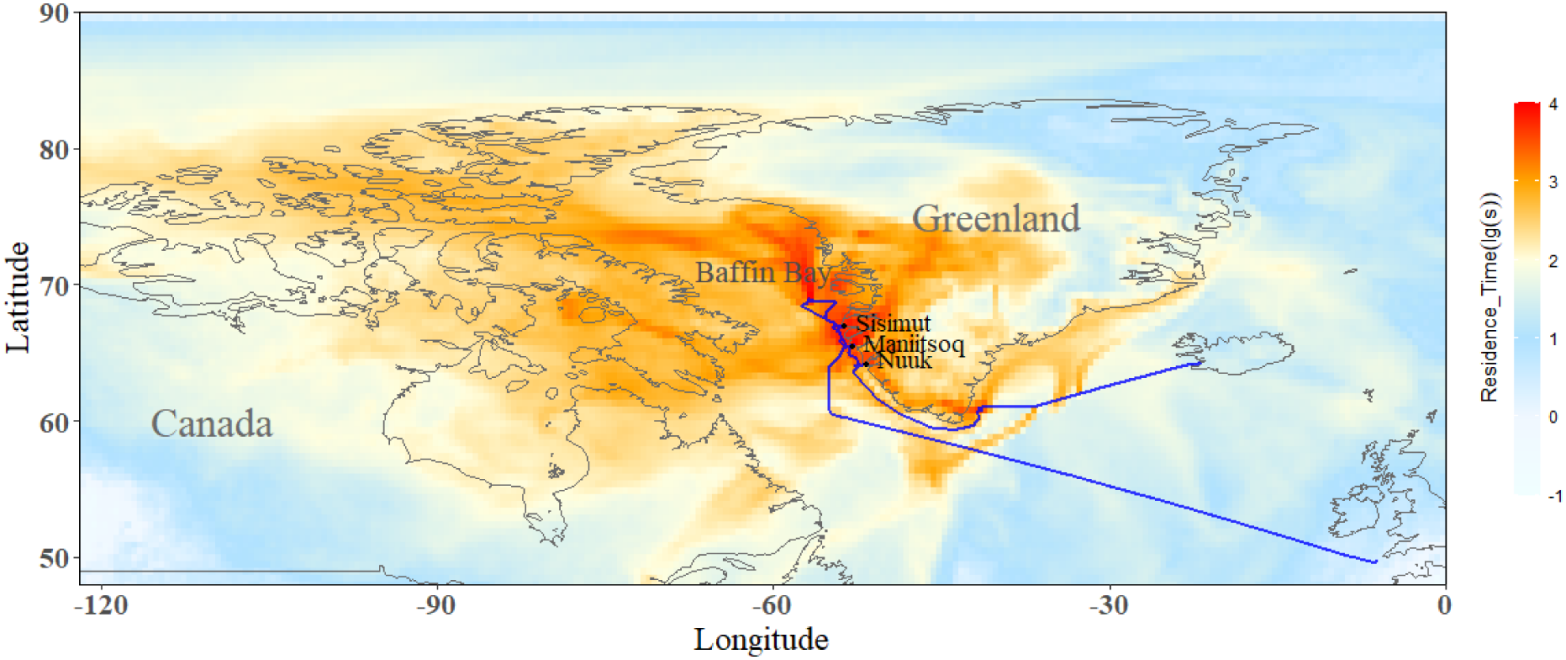
Back trajectory simulated by FLEXPART during our observation (from 24 May to 17 June 2022). We only present the backward trajectories when the sampling location is in the Arctic (>60°N). The blue line shows the ship track.

The spatial variation patterns of BC mixing state (Figure 2c) are similar to those of BC mass concentrations (Figure 1b). We observed a high number fraction of soot-containing aerosols in Greenland coastal regions (Figure S11a), half of which are externally mixed (Figure 2c) and are typically freshly emitted.^51,52^ In contrast, BC over the open sea and MIZ is predominantly internally mixed (Figure 2c). This result indicates that the background BC is from long-range transport, as BC typically becomes internally mixed during the aging process.^53^ FLEXPART back-trajectory analysis further shows that air masses along the cruise track had spent time over the Greenland west coast during the last 10 days, which is within the typical atmospheric lifetime of BC (Figure 3). The west coast of Greenland hosts most of the island’s urban settlements, including the capital, Nuuk, the second-largest city as well as being Greenland’ largest seaport, Sisimiut, and several smaller towns such as Maniitsoq and Kangerlussuaq (Figure 3).

Previous studies have suggested sources of BC as long-range transported forest fires from northern Canada^11^ and local ship emissions.^54^ However, in this campaign, satellite imagery shows that only a few fire hotspots in Canada overlapped with the FLEXPART back trajectories (Figure S6b). As a result, there is limited influence of long-range transport from forest fires during our cruise. Meanwhile, the aerosol mass spectrometer (AMS) measurements from our cruise did not reveal any significant signal associated with ship exhaust.^55^ Moreover, a clear BC mass concentration gradient from the coastal region to the remote MIZ would not have been observed if biomass burning had been the dominant source.

To assess the representativeness of our data for the Greenland emission source, we performed additional 10-day backward trajectory analyses using HYSPLIT for the same cruise track across 2019–2025 (Figure S19) and for four seasons in 2022 (Figure S21, winter, spring, summer, and autumn). Air masses consistently showed significant residence time over the Greenland west coast across all years and seasons, confirming that the Greenland source identified during our campaign reflects a climatologically robust feature of near-surface air mass transport in the marine boundary layer of our study, rather than an exceptional meteorological condition. Together, these results indicate that Greenland is likely the primary source of BC in the Arctic marine boundary layer.

### 4.3 Atmospheric Implications

Our results show that background BC concentration in the marine boundary layer between Greenland and Canada in early summer is among the lowest observed in the Arctic (Table S3). Our observed rBC in the coastal region (2.4 ng m^–3^) is similar to the modeled BC of 3–5 ng m^–3^ at Station Villum during May–June.^56^ However, the modeled BC is much lower than the observed eBC of 9–26 ng m^–3^ reported by the same study,^56^ suggesting that the eBC measurements failed to constrain modeled BC. Similarly, Massling et al.^45^ modeled BC at Station Nord during summer as 19 ± 13 ng m^–3^, which is close to their observed eBC (11 ± 9 ng m^–3^) and elemental carbon (20 ± 22 ng m^–3^), and also within the range of our observed eBC within the Arctic (5.7–17.7 ng m^–3^). All of these values, however, are substantially higher than our shipborne rBC observations over the sea surface (0.6–2.4 ng m^–3^). Because eBC tends to overestimate background BC concentrations, constraining models with these elevated values introduces systematic biases, which could result in an overestimation of Arctic background BC in global simulations. Accurate quantification of background BC is critical for assessing the impacts of anthropogenic perturbations in the Arctic, as an overestimated baseline can exaggerate its role^5^ and misguide mitigation strategies. Our measurements provide rare observational constraints of rBC in the western Arctic and underscore the need for targeted modeling and further observations to improve understanding of Arctic aerosol-climate interactions, particularly in poorly sampled regions such as Greenland.

Second, our study identified the role of the Greenland west coast as a local source of BC in the Arctic marine boundary layer, although the shipborne observation revealed low BC mass concentrations and a small BC mass fraction in PM_2.5_. Previous studies suggest that BC in Greenland is primarily from long-range transported biomass burning.^57,58^ Several studies even reported zero BC emissions in the Greenland region,^22,59,60^ likely leading to an underestimation of anthropogenic emissions in Greenland. Our results indicate that anthropogenic emissions from Greenland are small but can still contribute to BC in the Arctic.

Here, we emphasize that BC particles from within the Arctic can travel long distances to sea ice or snow (Figure 4). Once these BC particles are deposited on the ice surface, these optically absorbing aerosols might accelerate ice melting.^3^ During regional transport, these BC particles become aged and are internally mixed with sulfate, secondary organic aerosols, and aged sea salt (Figures 2 and 4). Sulfate is mainly formed from dimethyl sulfide (DMS) oxidation,^55^ and secondary organic aerosols (SOA) are derived from volatile organic compounds (VOC).^30^ These aging processes can change particle properties, such as their mixing state and hygroscopicity, which strongly enhance their optical absorption and cloud condensation nuclei (CCN) activity.^53^ In this study, we found some coarse particles containing BC that were internally mixed with coarse sea salt (Figure 2d). Once lifted into the upper troposphere, these particles can act as giant CCNs due to their sea salt content, allowing BC to be incorporated into cloud droplets and influence Arctic cloud properties.^4,61^ In addition, BC is a tracer of anthropogenic emissions, and anthropogenic pollutants can enhance the ice-nucleating particle (INP) activity of sea salt.^62^ Therefore, the observed internal mixing of BC with sea salt aerosols indicates that transported anthropogenic pollutants potentially enhance the INP activity of Arctic marine aerosols, further influencing polar cloud formation. These microphysical changes in individual BC particles should be considered in models to better evaluate their climate effects.

**Figure 4:**
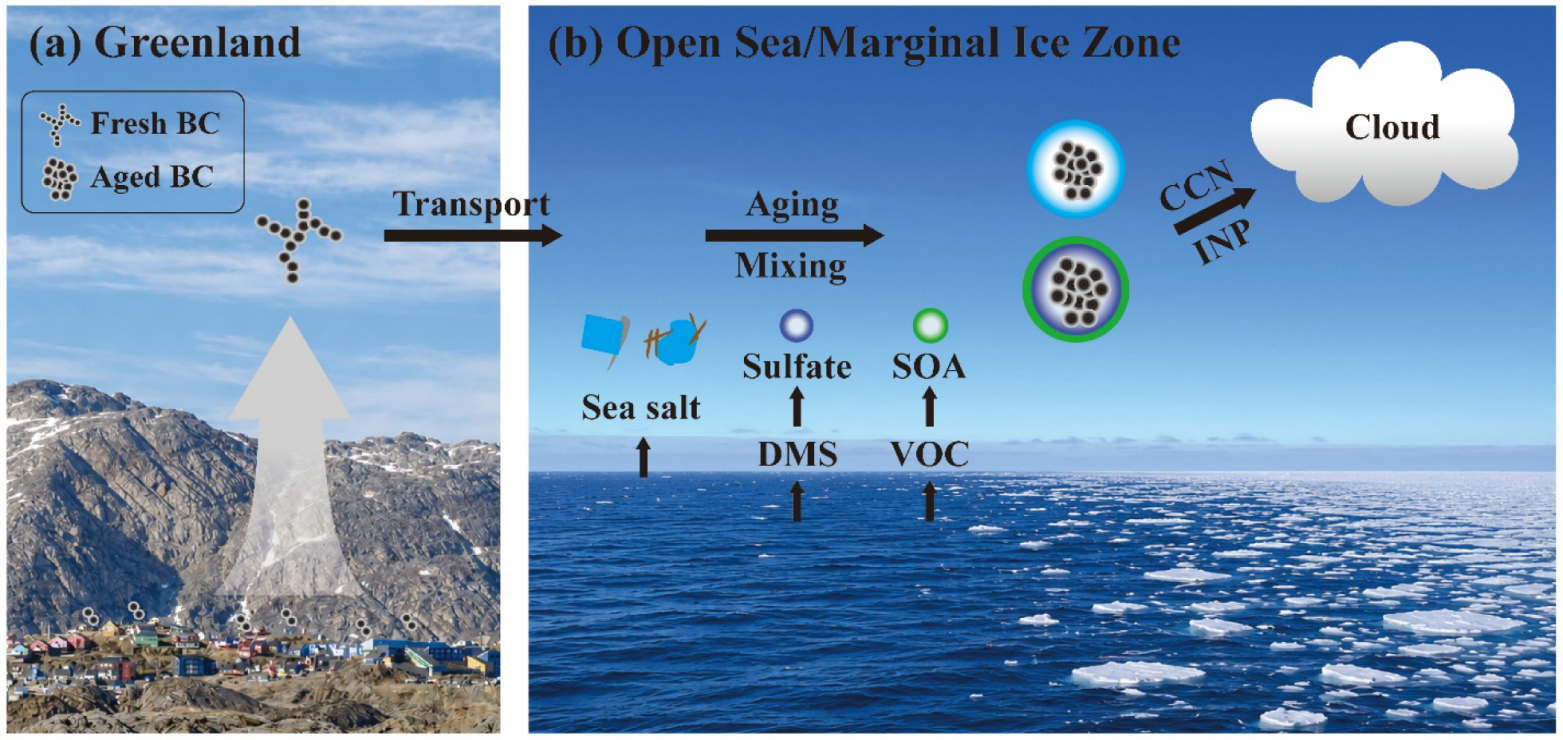
A schematic diagram of the emission and aging of BC in the Arctic. (a) In coastal regions, BC is emitted from residential activities, initially existing as chain-like aggregates. (b) During transport over the open sea, BC undergoes atmospheric aging and mixing processes. BC is internally mixed with aged sea salt and secondary aerosols, including sulfate formed from dimethylsulfide (DMS) oxidation and secondary organic aerosols (SOA) derived from volatile organic compounds (VOC). Aged BC-containing particles potentially enhancing their ability to act as cloud condensation nuclei (CCN) or ice crystal particles (INPs), thereby influencing cloud formation and regional radiative balance.

## Supplementary Material



## Notes

Details of campaign routine and sampling procedures; TEM particle classification methodology and individual particle sampling information; classification of air masses and back trajectory analysis; sea ice concentration processing and marginal ice zone identification; comparison of eBC and rBC mass concentrations and AE33 calibration procedures; size distributions of individual particles by type and air mass region; overview of Arctic BC observations at land sites and from shipborne campaigns; and representativeness analysis of Greenland emission contributions via multi-year and multi-season backward trajectory residence time frequency (PDF)

## ∇

Now at: National Centre for Atmospheric Science (NCAS), University of Manchester, Manchester, M13 9PL, UK
