# Supplementary material for: Greenland Emissions Contribute to Black Carbon Aerosols in the Arctic Marine Boundary Layer
Source: Environ Sci Technol. 2026 Jul 22;60(30):21124–34. doi: 10.1021/acs.est.6c03343 (PMC13449892; doi:10.1021/acs.est.6c03343)
Supplement: Supplementary file 1 [file es6c03343_si_001.pdf]

Supplementary Information for

# Greenland emissions contribute to black carbon aerosols in the Arctic marine boundary layer

*Xiaomi Teng[1], Mao Du[2], Congbo Song[2, a], Yuqing Dai[2], Vipul Lal Chandani[2], David C. S. Beddows[3], Manuel Dall'Osto[4], Darrel Baumgardner[5], Roy M. Harrison[2], James Brean[2], Mingxi Yang[6], Yangmei Zhang[7], Weijun Li[1, *], and Zongbo Shi[2, *]*

[1] Department of Atmospheric Sciences, School of Earth Sciences, Hangzhou, 310058, Zhejiang University

[2] School of Geography Earth and Environmental Sciences, University of Birmingham, Birmingham, B15 2TT, UK

[3] National Centre of Atmospheric Science, School of Geography, Earth & Environmental Sciences, University of Birmingham, Edgbaston, Birmingham, B15 2TT, UK.

[4] Department of Marine Biology and Oceanography, Institute of Marine Sciences (ICM, CSIC), Barcelona, 08003, Spain

[5] Droplet ENVEA Group, Longmont, CO, 80503, USA

[6] Plymouth Marine Laboratory, Prospect Place, West Hoe Plymouth, PL1 3DH, UK.

[7] State Key Laboratory of Severe Weather/Key Laboratory of Atmospheric Chemistry of China Meteorological Administration, Chinese Academy of Meteorological Sciences, Beijing 100081, China

[a] Now at: National Centre for Atmospheric Science (NCAS), University of Manchester, Manchester M13 9PL, UK

Correspond to: Weijun Li (liweijun@zju.edu.cn); Zongbo Shi (z.shi@bham.ac.uk)

## Contents

**S1 Campaign routine** ........ 3

**S2 TEM measurements** ........ 3

**S3 Classification of sampling air mass** ........ 7

**S4 Comparison of eBC and rBC mass concentration** ........ 9

**S5 size distribution of individual particles** ........ 10

**S6 AE33 calibrations** ........ 12

**S7 Overview of the land observation station and the ship cruise track in the Arctic.** ........ 14

**S8 representativeness of Greenland emission** ........ 20

**Reference** ........ 22

## S1 Campaign routine

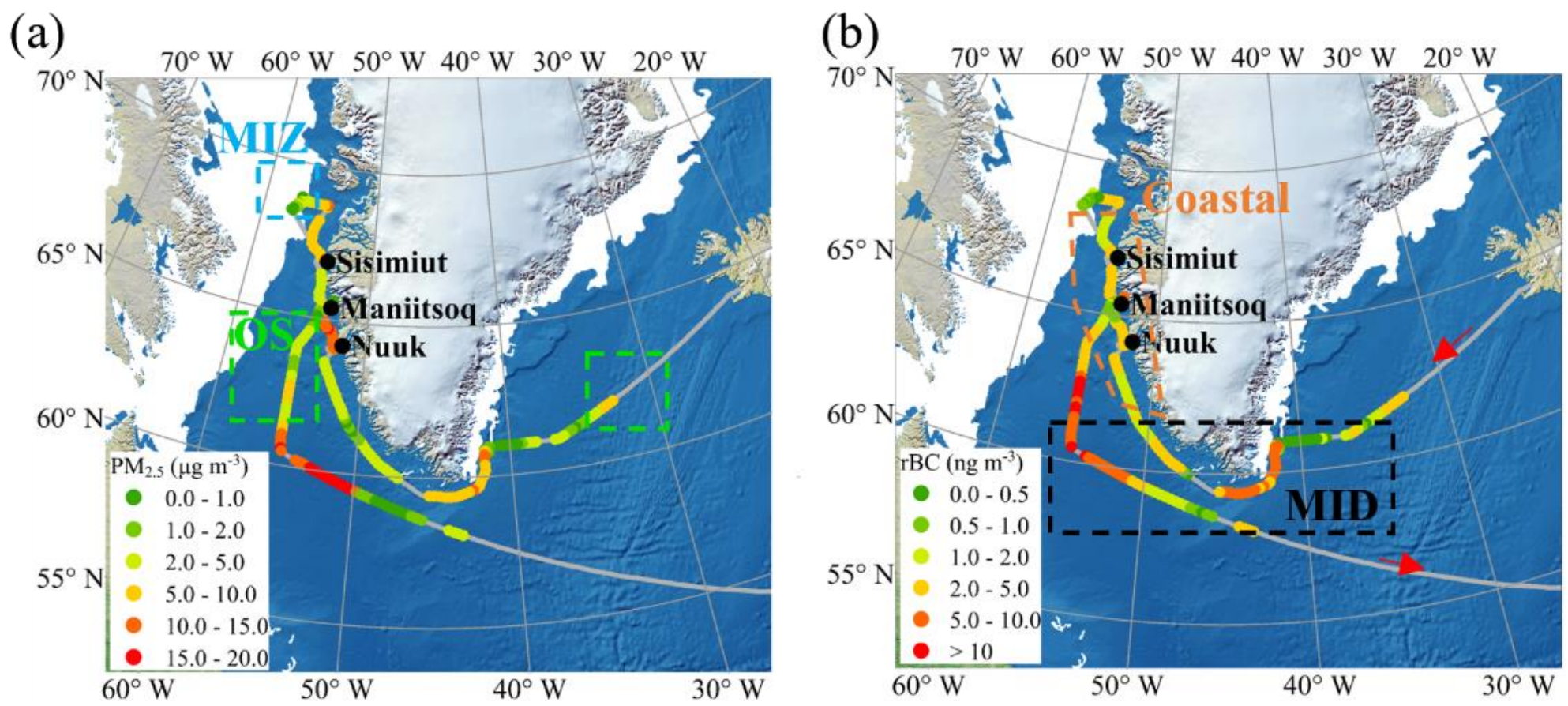


**Fig. S1 (a)** Geographical distribution of $PM_{2.5}$ mass concentrations along the cruise track. White shading indicates sea ice cover on 12 June, based on data from the National Snow and Ice Data Center (NSIDC; https://nsidc.org/data, last access: 4 July 2023). Map from Natural Earth (https://www.naturalearthdata.com/, last access: 17 October 2024). **(b)** Geographical distribution of refractory black carbon (rBC) mass concentrations along the cruise track. Red arrows show the navigation direction.

## S2 TEM measurements

### Text S1 TEM classification

Particles were classified into five major types based on morphology and elemental composition obtained from TEM-EDS: (1) soot-containing particles, identified by their distinct morphology that is chain-like or grape-like aggregates; (2) mineral dust, characterized by irregular morphology and Si-, Al-, Ca-, or Fe-rich compositions (Al > 1 wt% and Fe > 1 wt% or Si >1wt%); (3) sea salt particles, identified by Na-bearing composition (Na > 1 wt%); (4) S-rich particles, identified by sulfate-bearing composition and bubble-like sphere; and (5) other particles, including metals, fly ash, and organic particles. Figure S1 shows the flow chart describing the classification scheme. When particles included two or more components (e.g., mineral dust mixed with sea salt), they were classified in the upper category, which was in the order of soot-containing particles > mineral dust particles > Sea Salt particles > S-rich particles >other particles. The classification obtained using TEM-EDS has the disadvantage of potentially misclassifying; for example, highly aged Sea Salt particles may be highly similar to sulfate particles. On the other hand, an advantage of this classification is that it can separate soot-containing particles from other particles, along with the shapes and mixing states of measured particles. Figure S3 shows the representative EDX spectra and TEM images for each particle class.

**Table S1** Sampling information of individual particles.

| Type | Sample ID | Sampling time (UTC) | Sampling site |
|---|---|---|---|
| Coastal | D4 | 2022/05/25 10:00-11:00 | East of Greenland |
| | G3 | 2022/05/28 12:30-13:30 | Approaching Nuuk |
| | J1 | 2022/05/31 10:00-11:10 | Leaving Nuuk |
| | P2 | 2022/06/05 13:30-14:50 | West Greenland Coast |
| | 2-J5 | 2022/06/15 01:00-02:20 | Near Maniitsoq |
| OS | F3 | 2022/05/27 09:00-10:00 | West of Greenland |
| | F5 | 2022/05/27 12:30-13:30 | West of Greenland |
| | 2-N3 | 2022/06/16 12:47-14:26 | West of Greenland |
| MIZ | 2-D3 | 2022/06/11 00:30-01:30 | Near the melting sea ice |
| | 2-D5 | 2022/06/11 15:25-16:45 | |
| | 2-F3 | 2022/06/12 14:00-15:20 | |

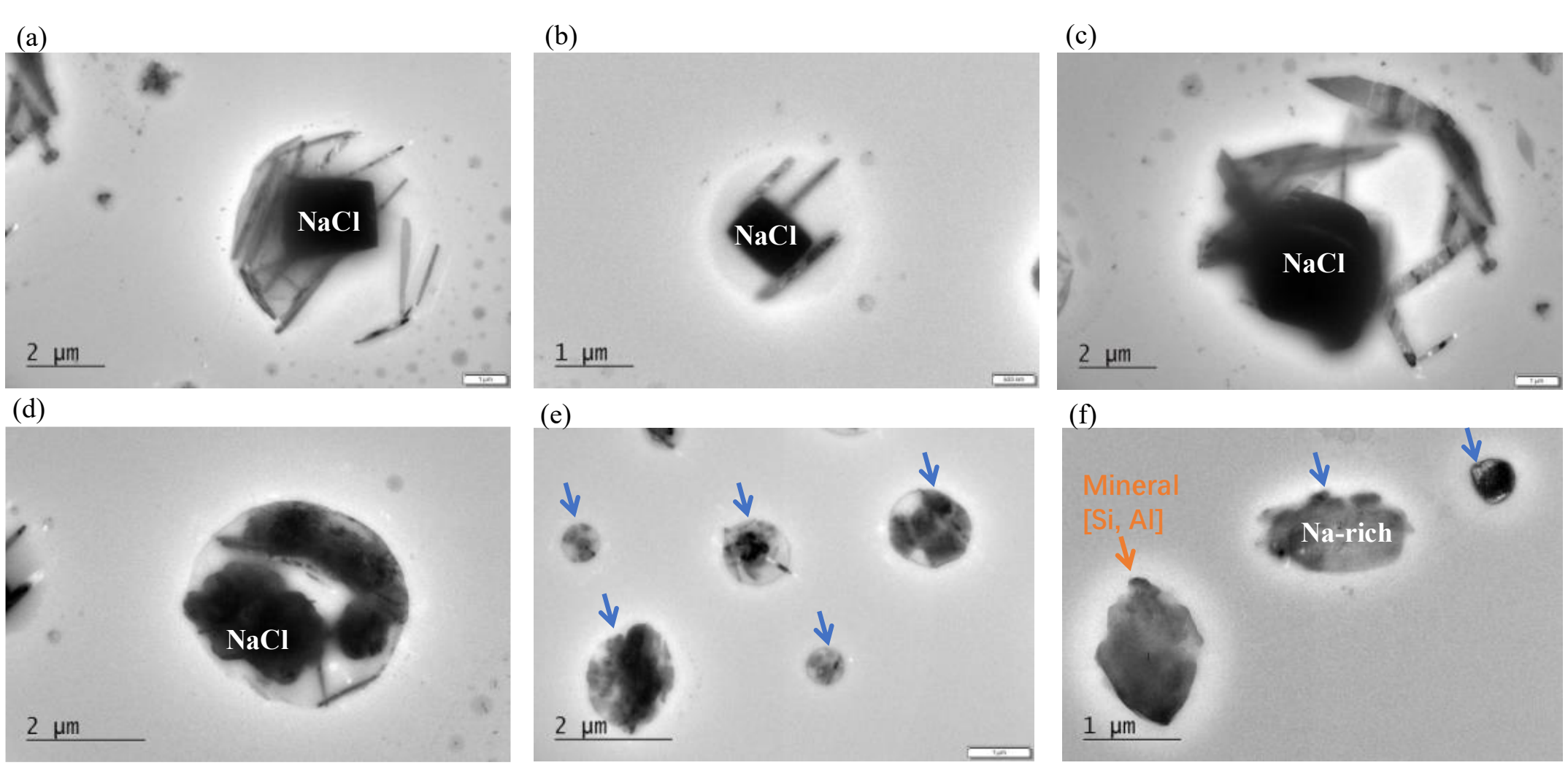


**Fig S2** The TEM images of sea salts in coarse stage of the individual particle sampler.

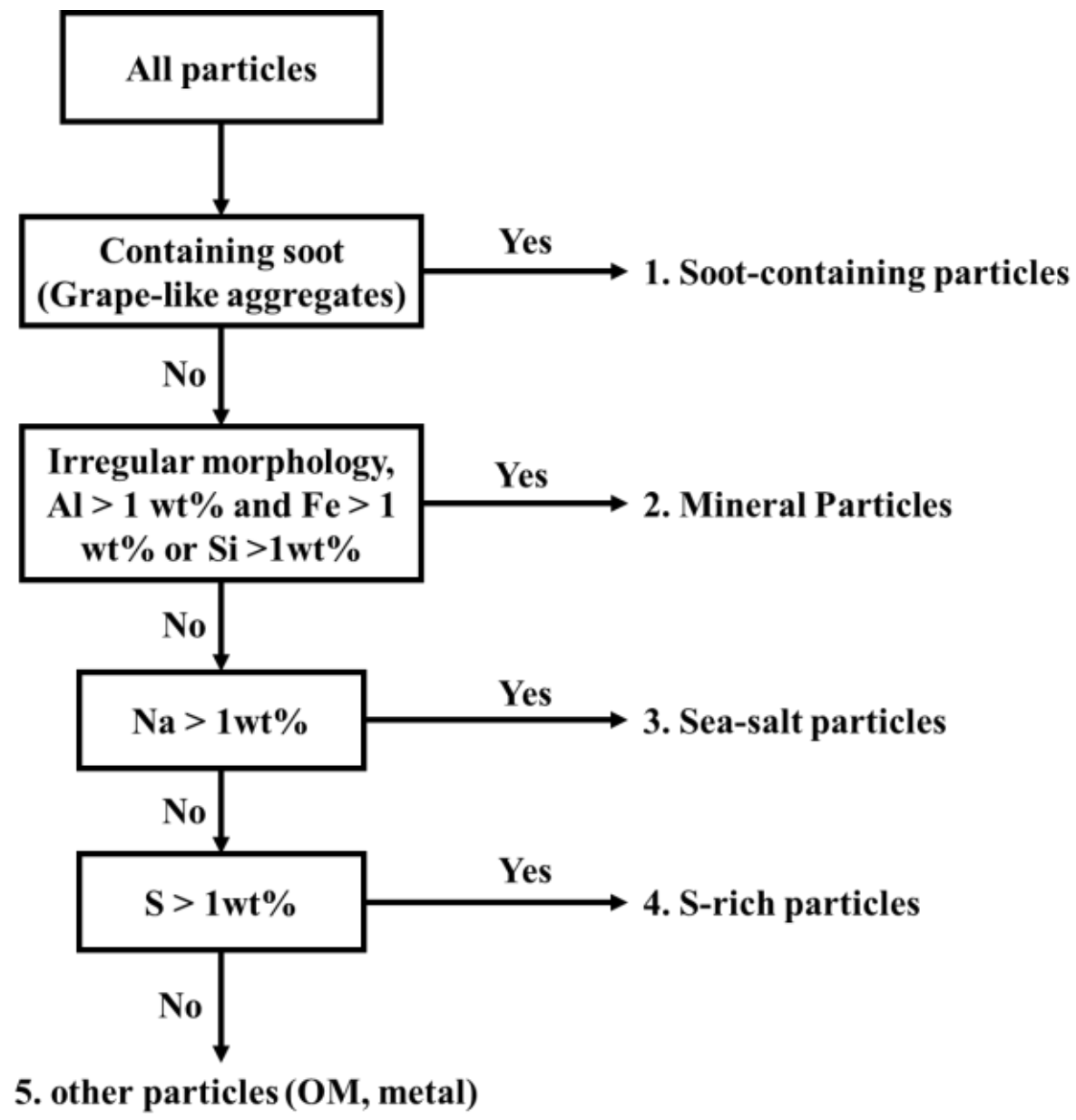


**Fig. S3** Flow chart to classify the individual particles into 5 categories based on the TEM-EDS analysis.

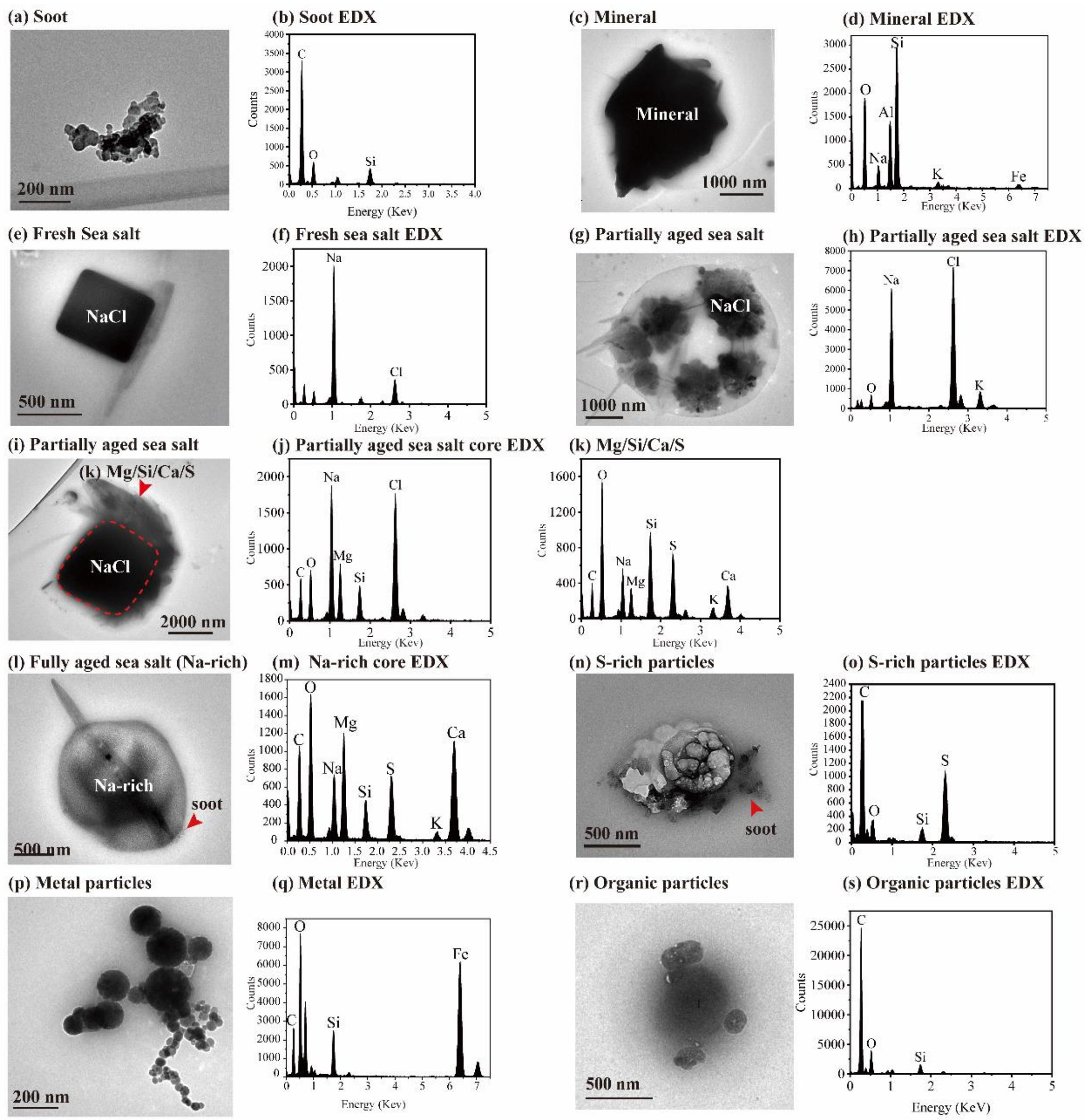


**Fig. S4** TEM images and EDX spectra of different particle classifications.

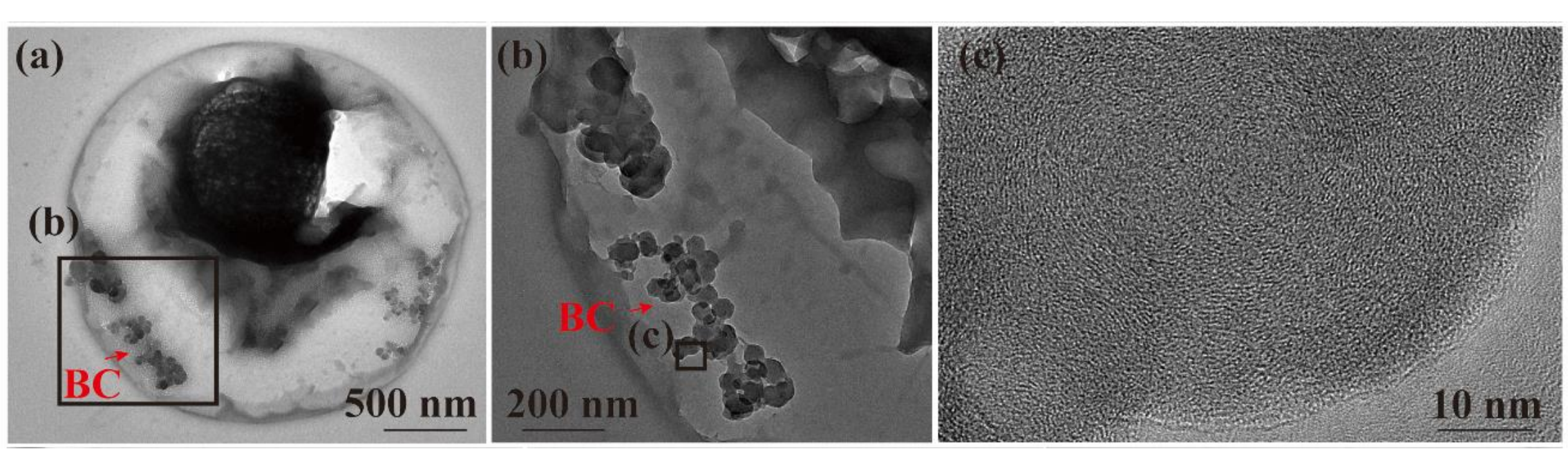


**Fig. S5** High-resolution images of BC particles.

## S3 Classification of sampling air mass

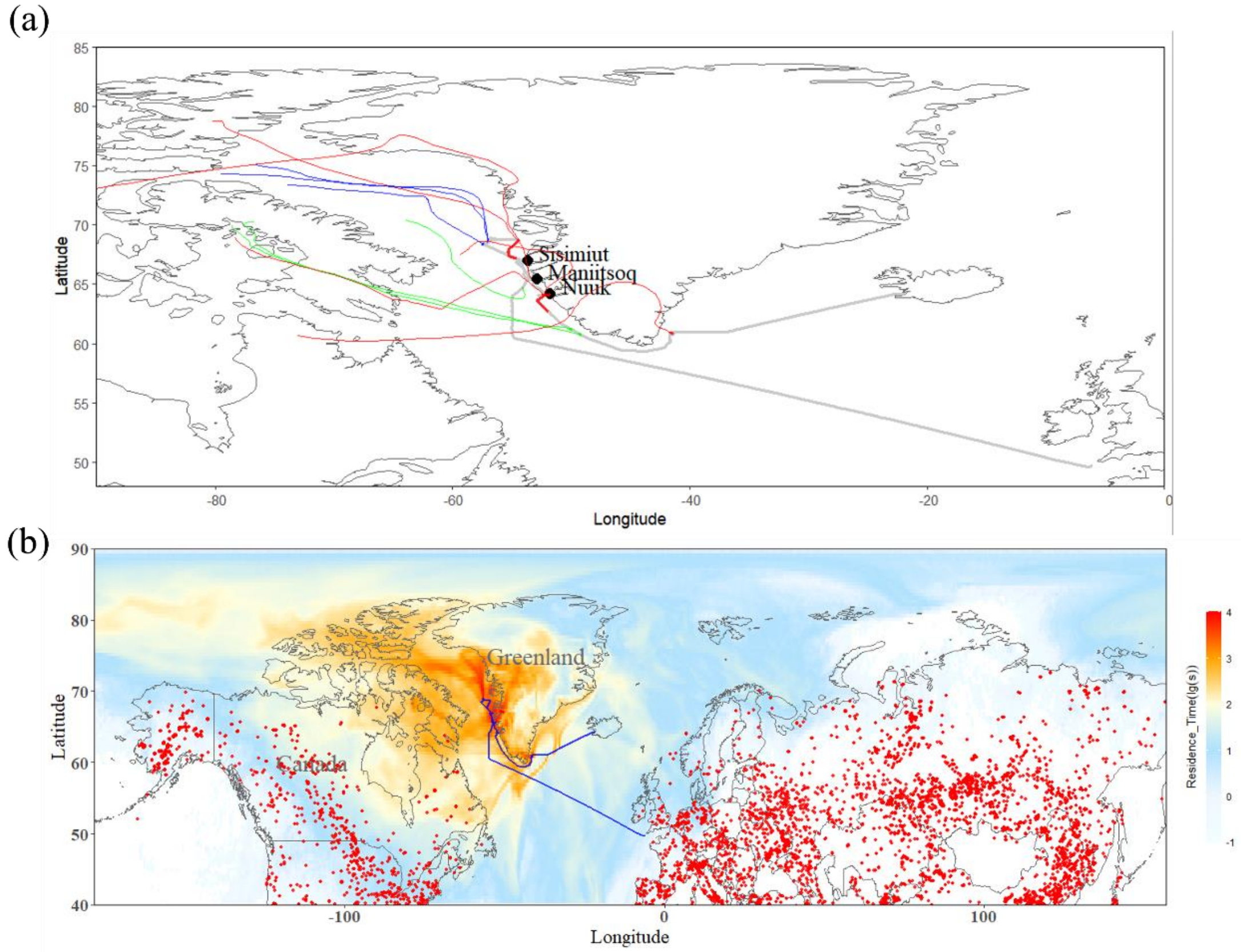


**Fig. S6 (a) The route of the cruise, the sampling locations, and 72 h back trajectories of analyzed TEM samples.** Nuuk is the capital of and the most populous city in Greenland. Maniitsoq is a town with about 2,534 inhabitants (2020, Wikipedia). Sisimiut is the second-largest city in Greenland, is the largest business center north of the national capital of Nuuk, and is one of the fastest-growing cities in Greenland. Colored lines are 72 h backward trajectories of TEM samples of coastal region (red), open sea (green), Marginal ice zone (blue). **(b) The back trajectory simulated by FLEXPART across the fire area in northern Canada during our observation (from 22 May to 19 June 2022).** We only present the backward when the sampling location in the Arctic (>60ºN). Fire data was downloaded from the Fire Information for Resource Management System, monitored by MODIS (https://firms.modaps.eosdis.nasa.gov/, last access: 22 Aug 2024).

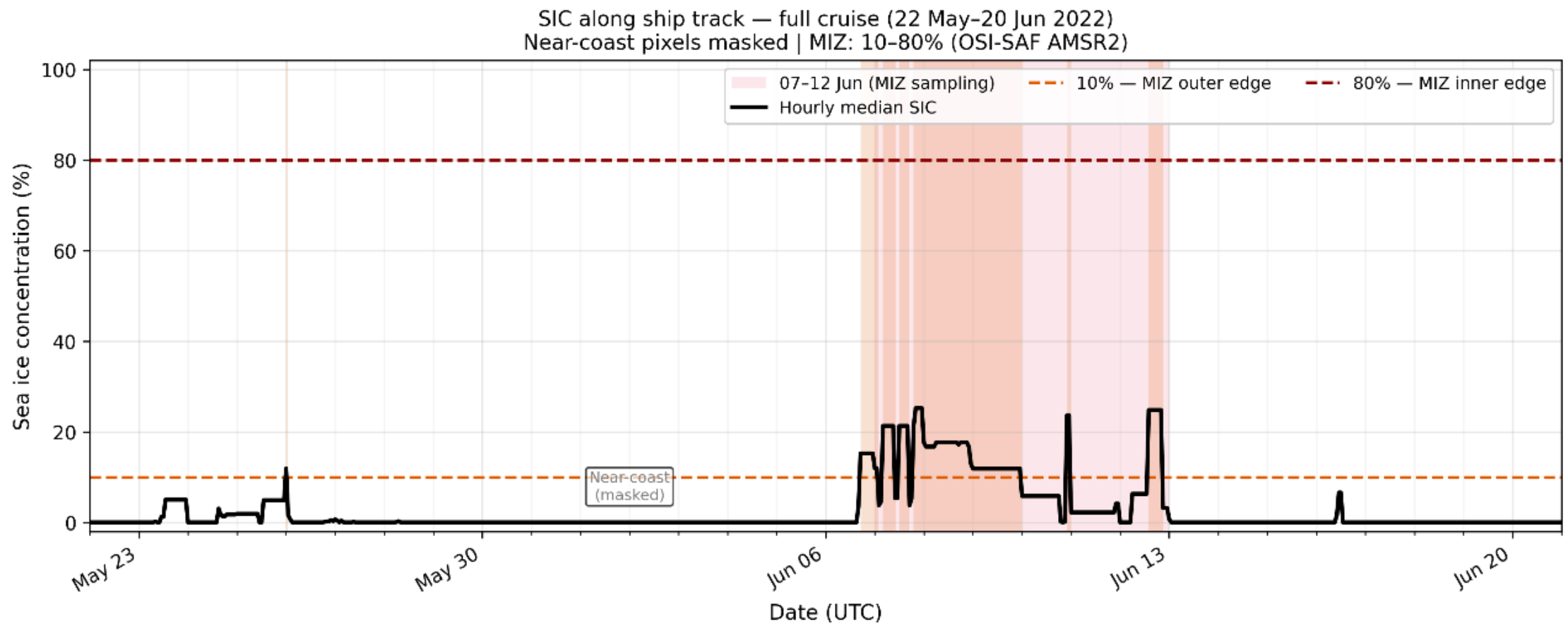


**Fig. S7** Time series of sea ice concentration (SIC) extracted along the ship track for the full cruise period (22 May–20 Jun 2022), based on daily OSI-SAF AMSR2 SIC fields (10 km resolution). The black line denotes the hourly median SIC at the ship's position. Orange and dark-red dashed lines indicate the 10% and 80% MIZ thresholds, respectively. The red shading marks the MIZ sampling period (7–12 June). Near-coast grid cells (within one pixel of land) are masked to avoid land-contamination artefacts in the passive microwave retrievals. A brief exceedance of the 10% threshold near Cape Farewell (~26 May) is excluded from MIZ classification, as the ice was attached to the coastal landmass rather than connected to the inner pack ice.

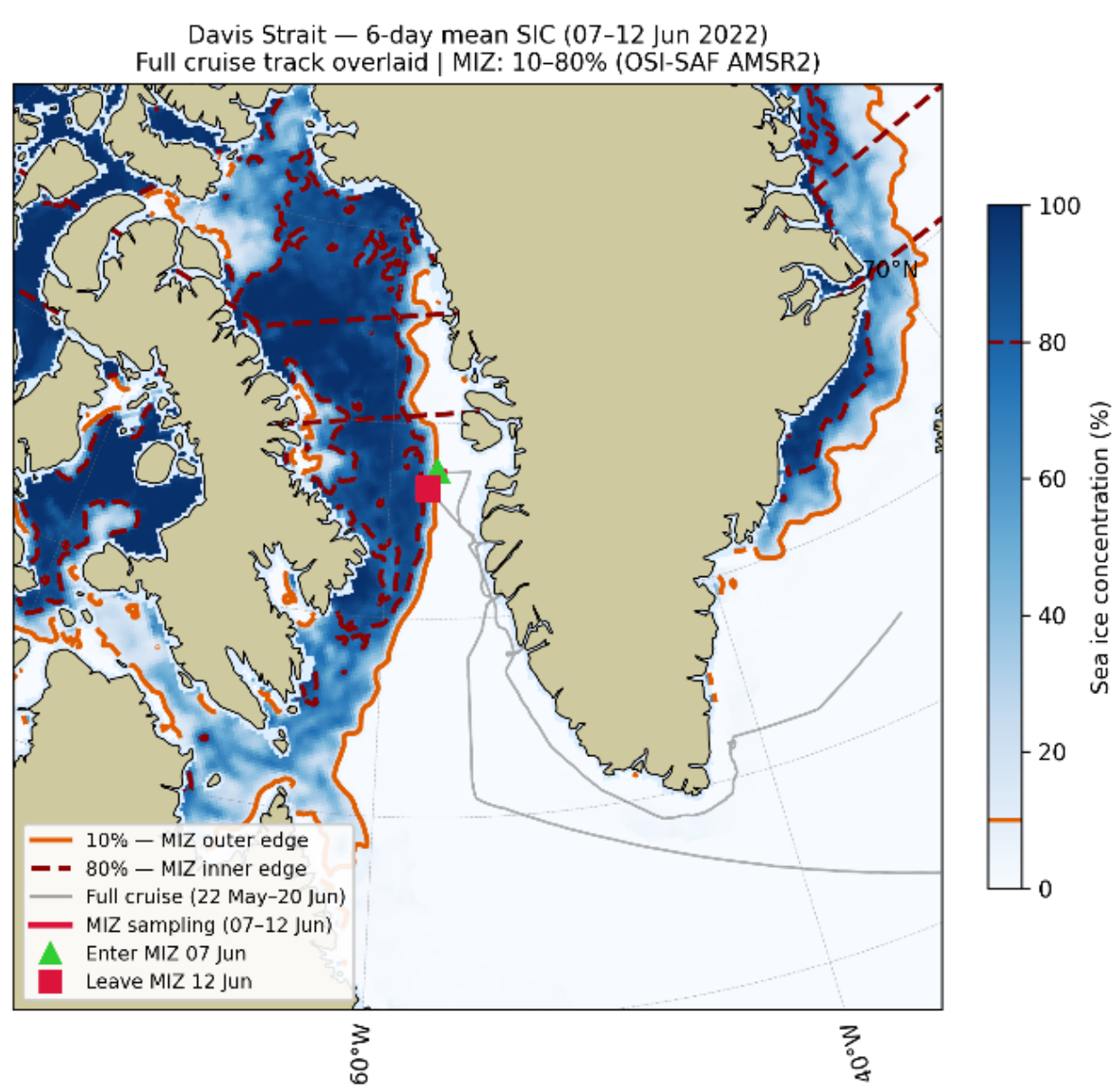


**Fig. S8** Six-day mean sea ice concentration (SIC) over the Davis Strait region (7–12 June 2022), derived from OSI-SAF AMSR2 passive microwave retrievals (10 km resolution). Orange solid and dark-red dashed contours indicate the 10% and 80% SIC thresholds, respectively, which define the outer and inner boundaries of the Marginal Ice Zone (MIZ). The full cruise track (22 May–20 Jun 2022) is shown in gray, with the MIZ sampling period (7–12 June) highlighted in red. The green triangle and red square mark the entry and exit points of the MIZ segment, respectively.

**S4 Comparison of eBC and rBC mass concentration**

**Table S2** Summary of aerosol properties showing the median and 1 standard deviation.

| | MID | | Coastal | | OS | | MIZ | |
|---|---|---|---|---|---|---|---|---|
| | Median | Standard Deviation | Median | Standard Deviation | Median | Standard Deviation | Median | Standard Deviation |
| $PM_{2.5}$ (μg $m^{-3}$) | 5.7 | 5.8 | 5.3 | 3.7 | 2.6 | 1.5 | 1.3 | 0.7 |
| rBC (ng $m^{-3}$) | 5.3 | 3.7 | 2.4 | 2.1 | 1.7 | 1.1 | 0.6 | 0.4 |
| eBC (ng $m^{-3}$) | 32.4 | 20.0 | 18.2 | 8.7 | 11.6 | 6.1 | 5.3 | 4.0 |
| rBC/$PM_{2.5}$ (%) | 0.06 | 0.11 | 0.04 | 0.15 | 0.07 | 0.08 | 0.05 | 0.07 |
| eBC/rBC | 5.8 | 3.9 | 6.8 | 12.3 | 6.5 | 11.9 | 8.5 | 6.1 |

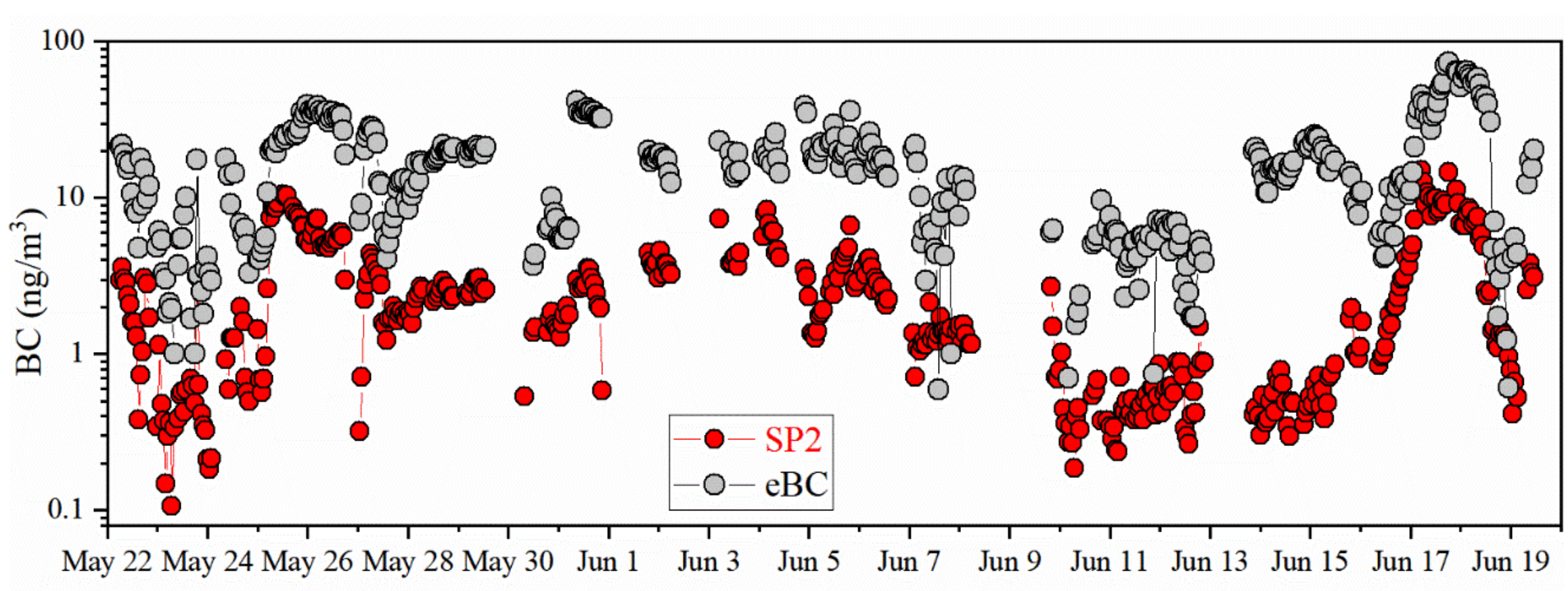


**Fig. S9** Comparison of eBC mass concentration measured using AE33 and rBC measured using SP2.

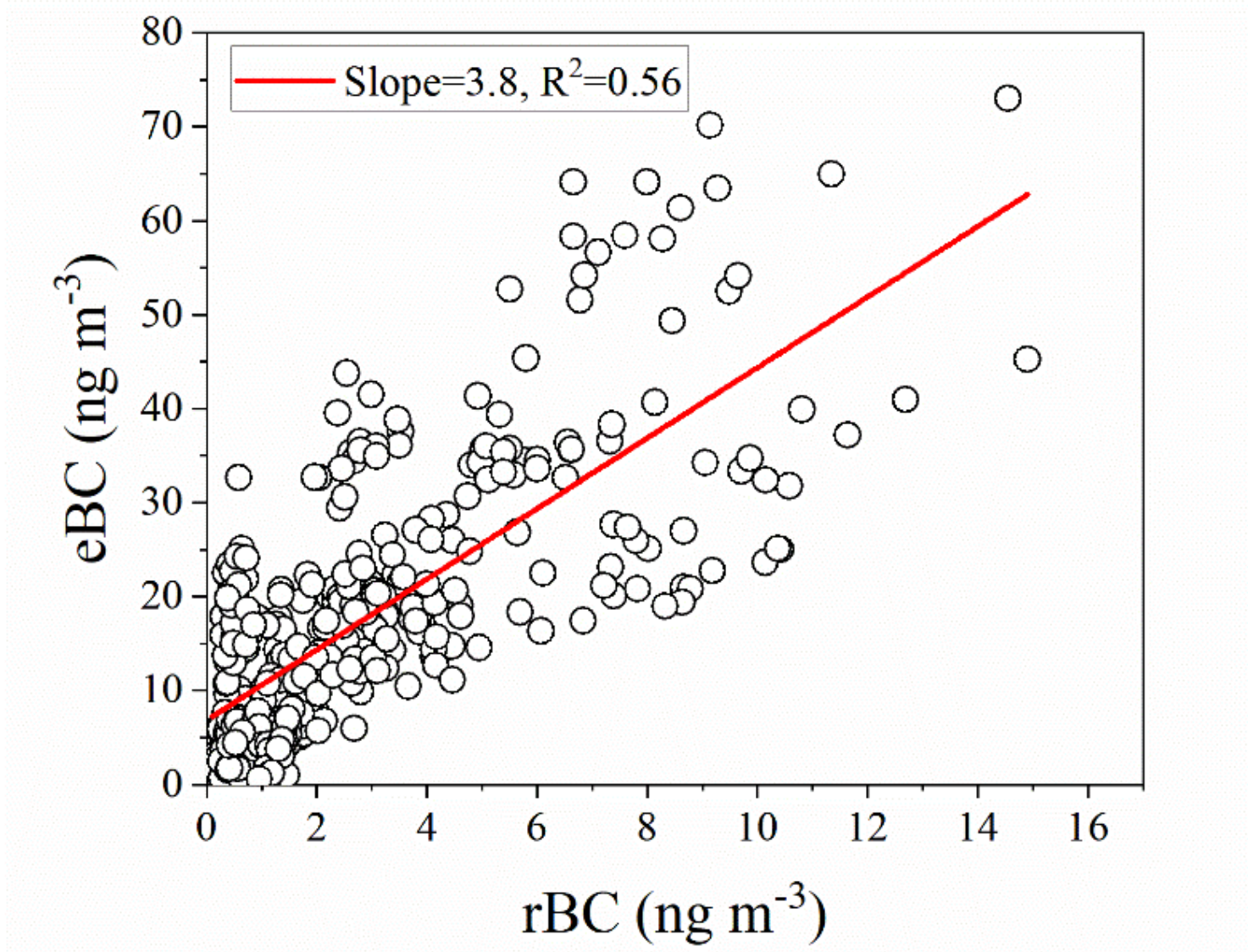


**Fig. S10** The x-y plot of eBC and rBC mass concentration.

**S5 size distribution of individual particles**

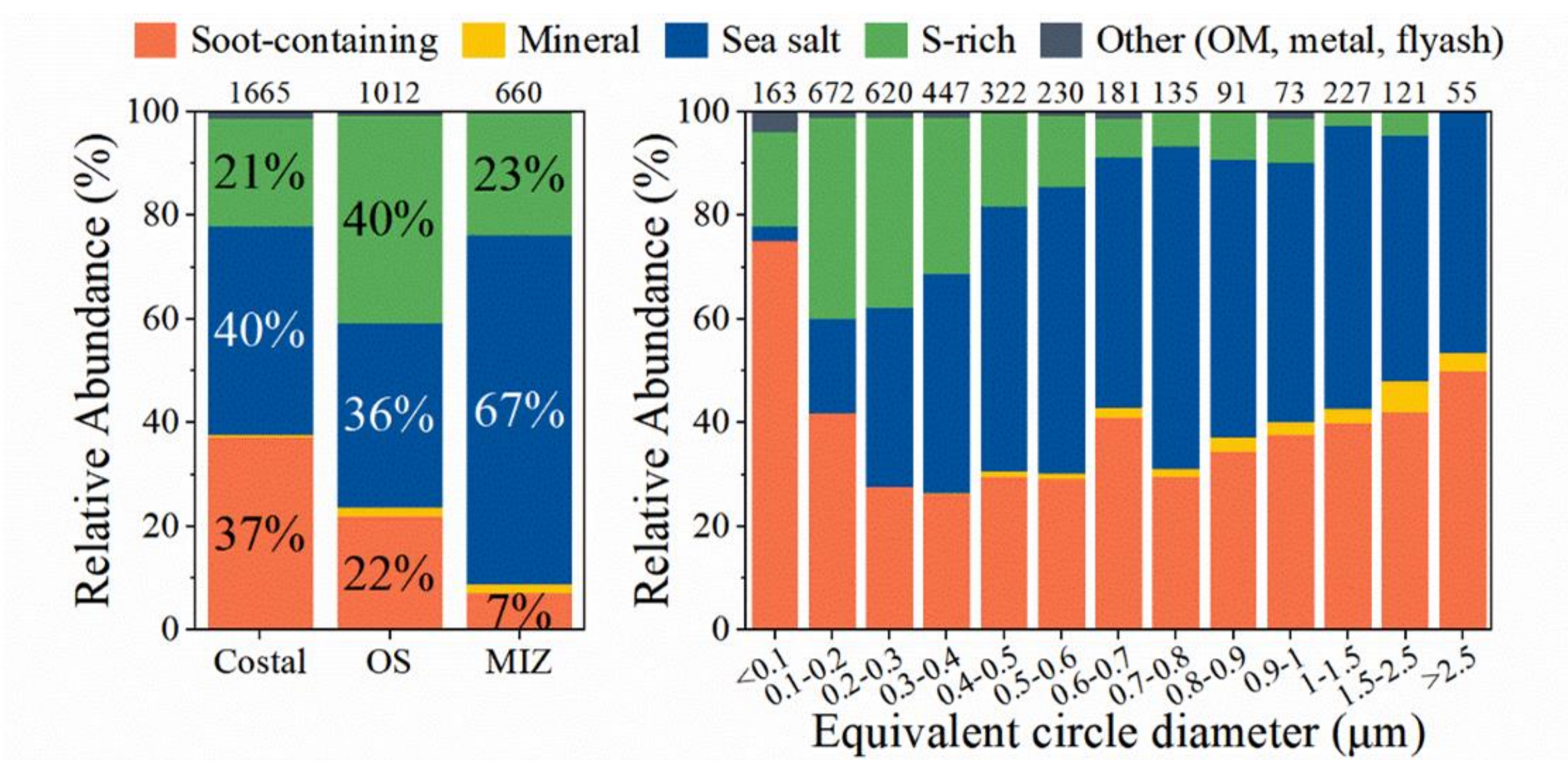


**Fig S11** The number fraction and size distribution of all particle types.

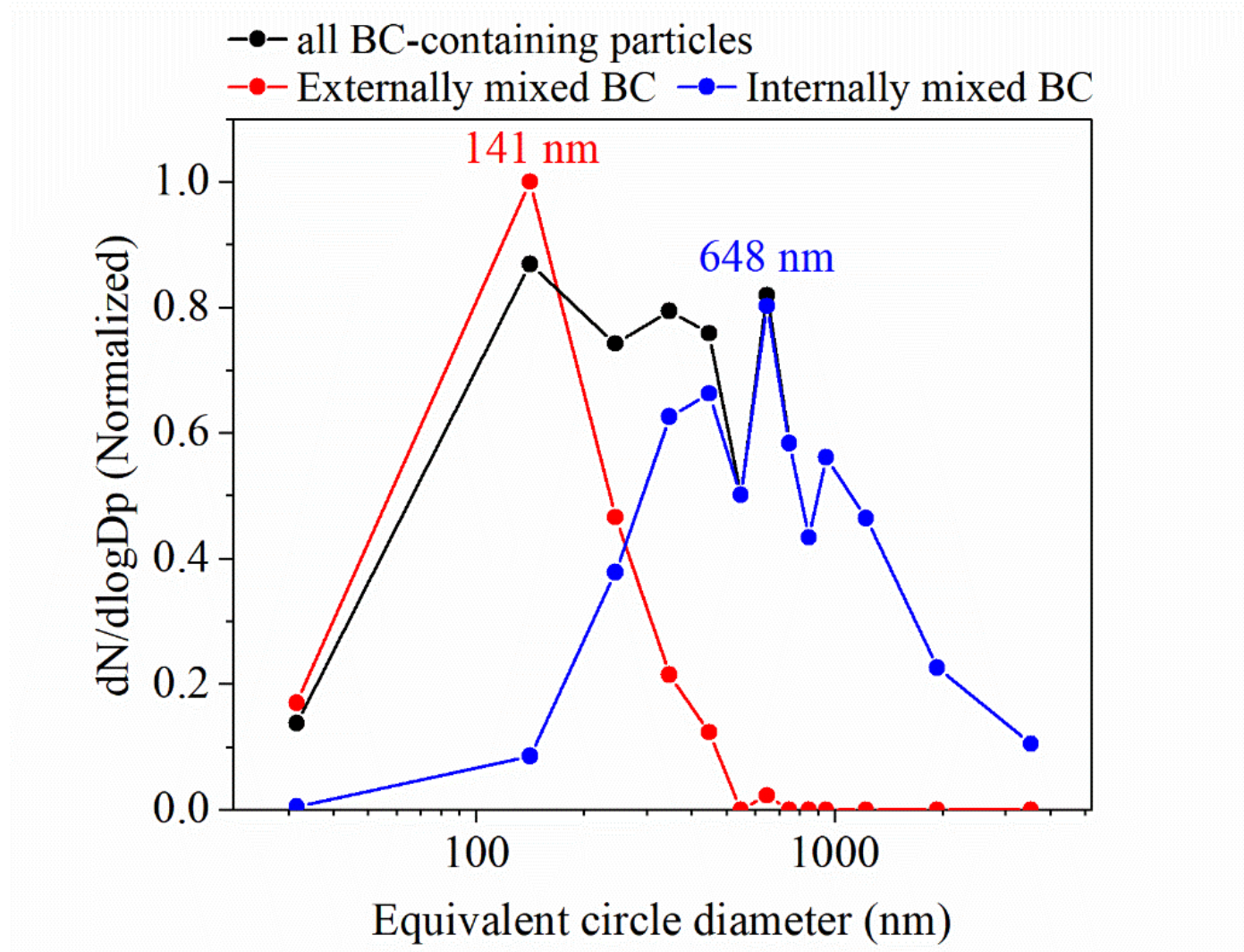


**Fig. S12** The size distribution of soot particles.

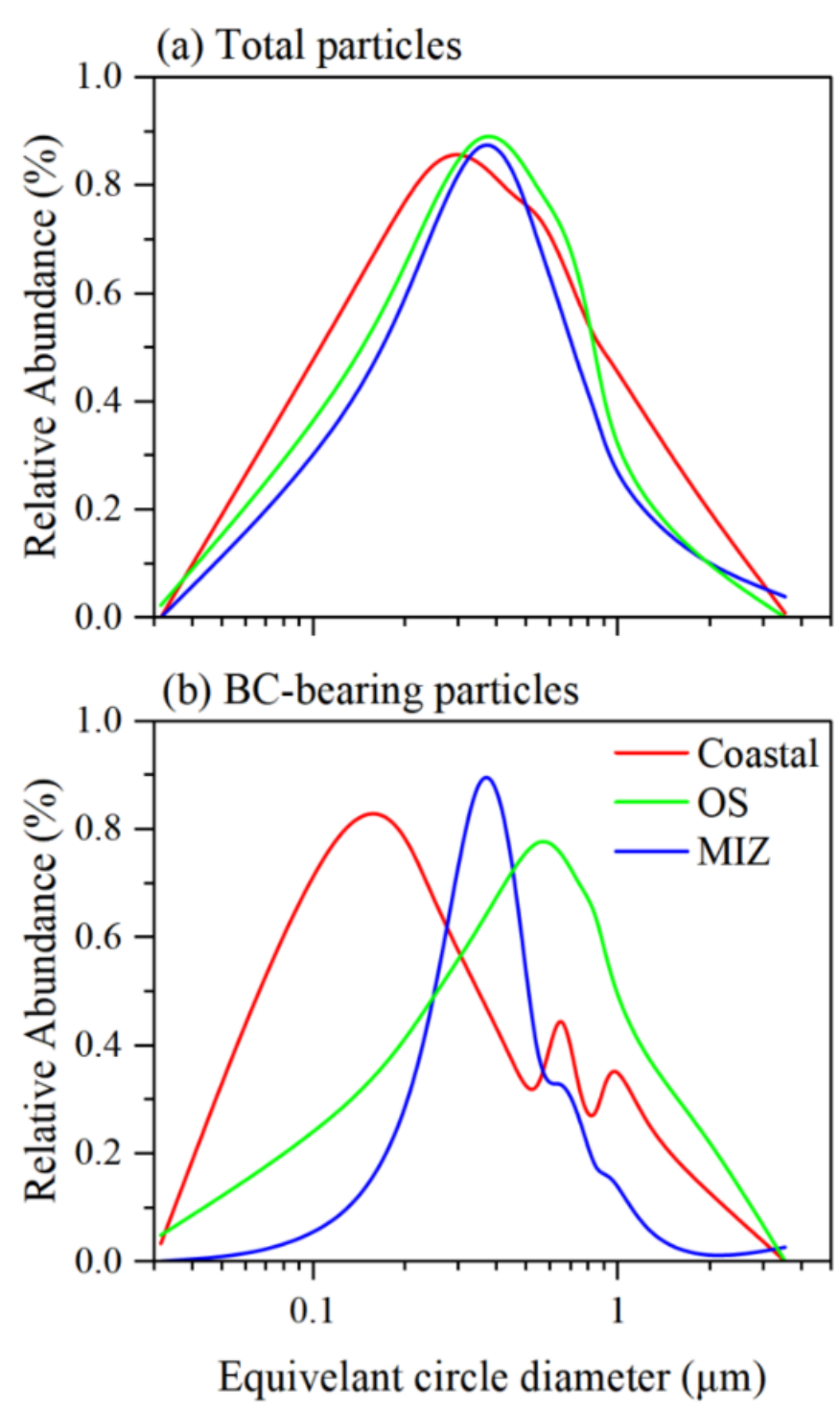


**Fig. S13** Size distributions of **(a)** total particles and **(b)** BC-bearing particles.

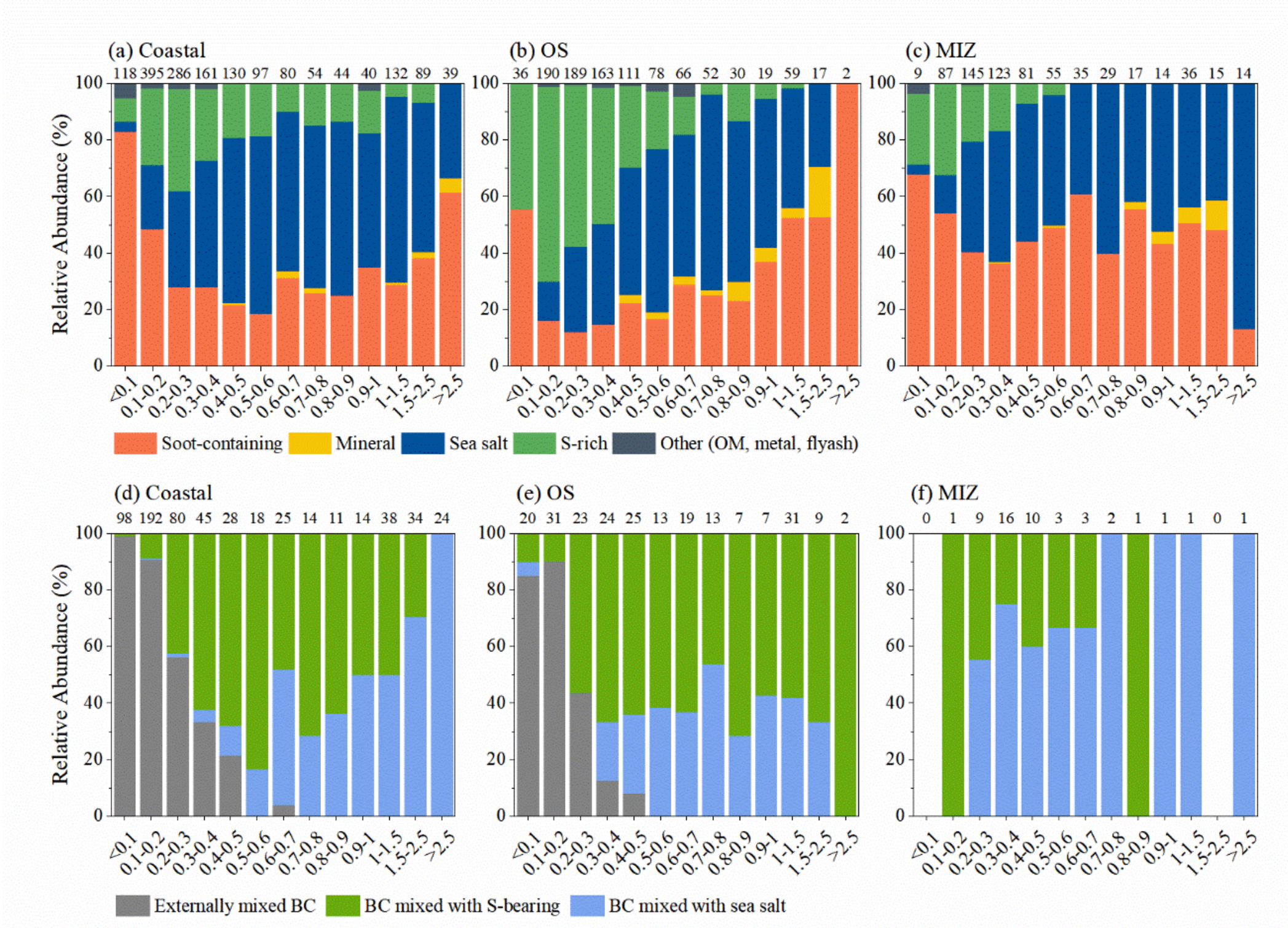


**Fig. S14 (a-c)** The size distribution of individual particles in coastal region, open sea, and MIZ. **(d-f)** The size distribution of individual BC particles in coastal region, open sea, and MIZ.

## S6 AE33 calibrations

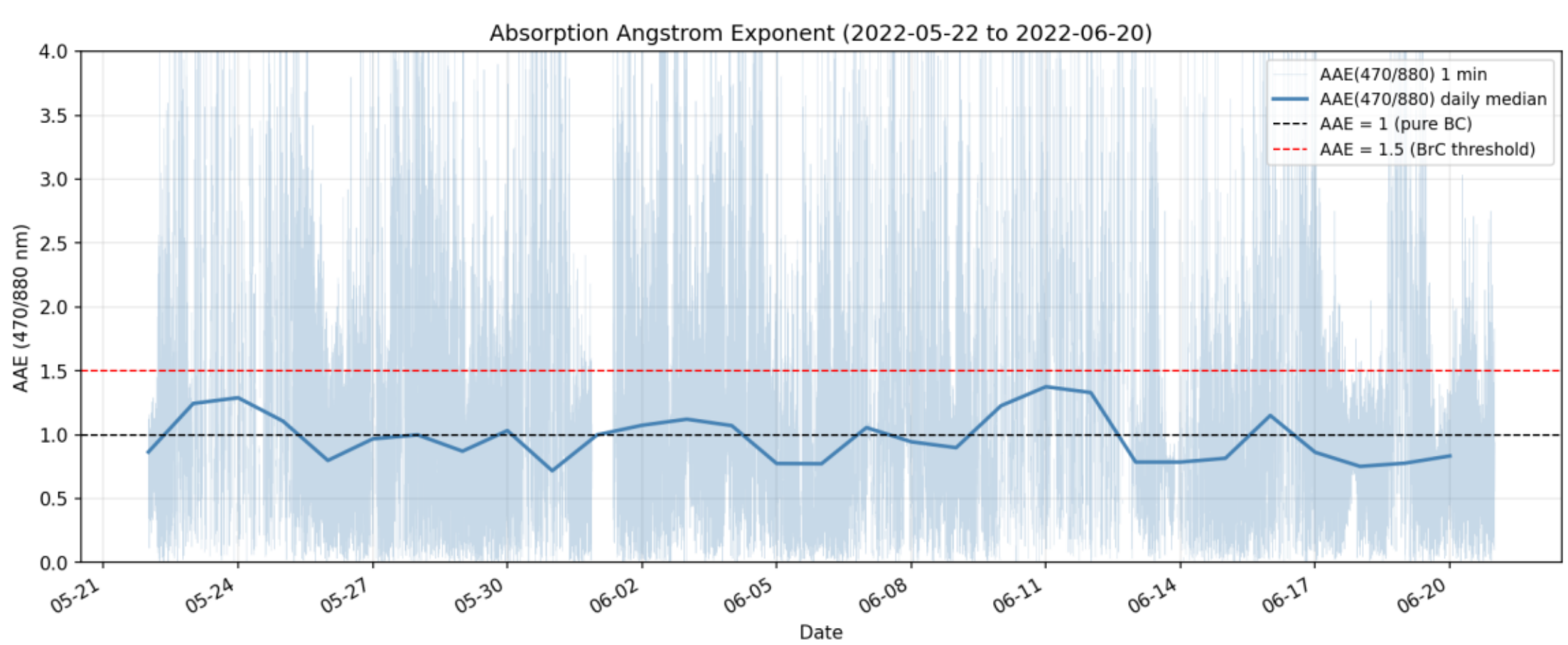


**Fig. S15** Timeseries of AAE (470/880 nm) during the campaign.

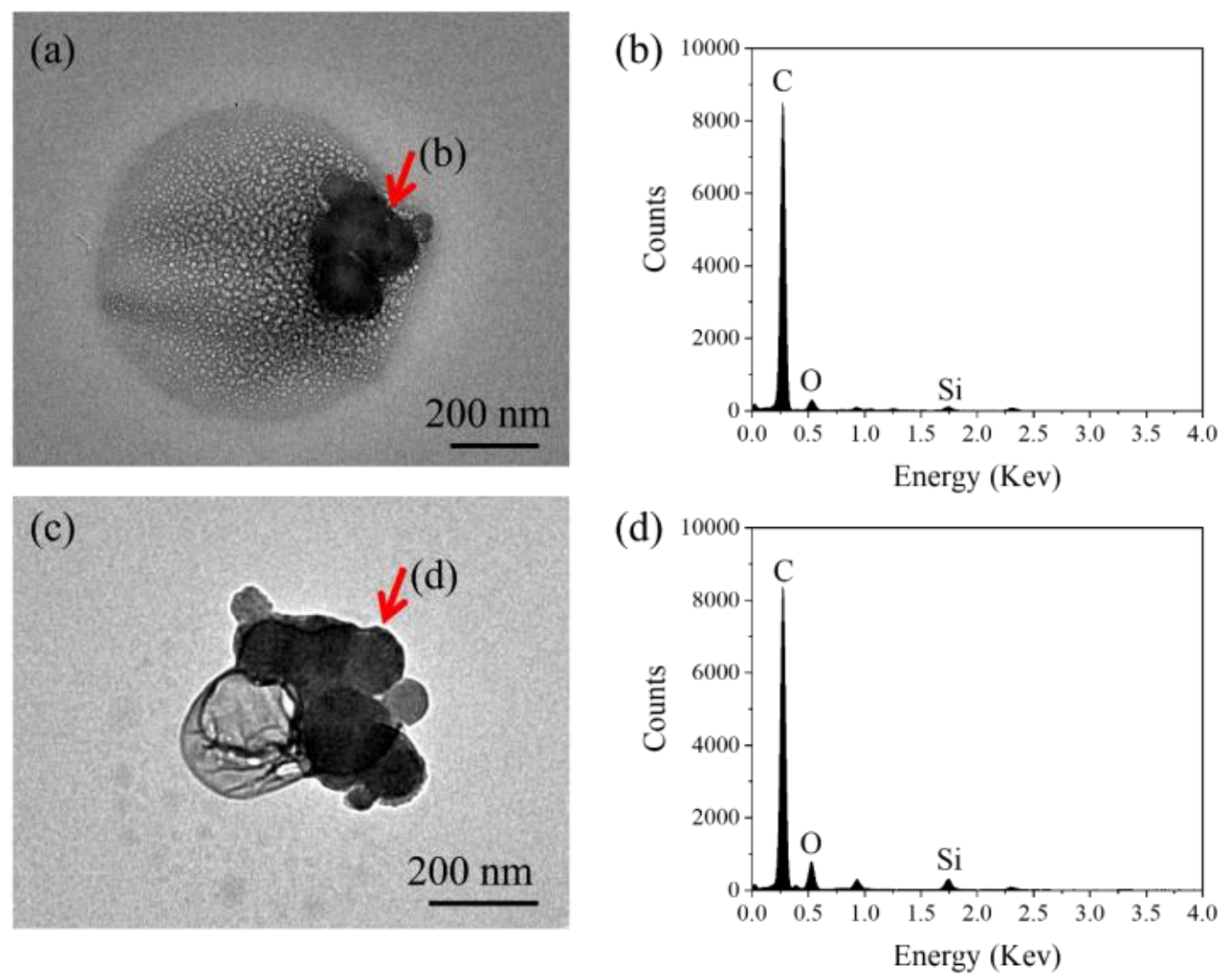


**Fig. S16** TEM images and EDX spectra of all tar ball aggregates observed during the cruise.

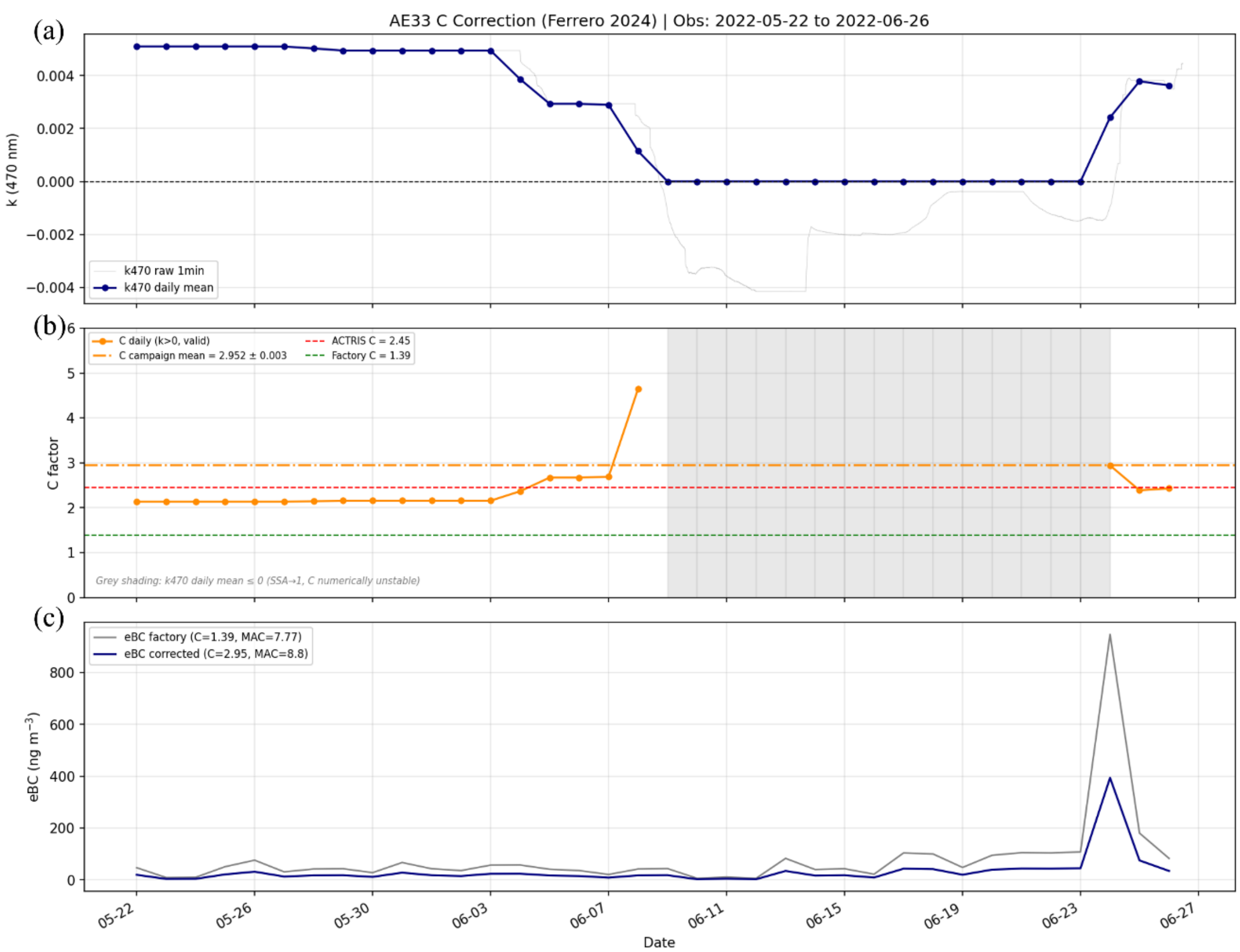

**Fig. S17** Time series of AE33 Aethalometer data correction parameters and equivalent black carbon (eBC) concentrations during the observation period (22 May–26 June 2022). **(a)** Daily mean (filled circles) and 1-min raw (grey line) filter loading parameter k at 470 nm. **(b)** Dynamically determined multiple-scattering correction factor C (orange circles, valid days only where k > 0), campaign-mean C = 2.952 ± 0.003 (orange dash-dot line), the Aerosol, Clouds and Trace gases Research InfraStructure (ACTRIS)-recommended value C = 2.45 (red dashed line; Yus-Díez et al., 2021), and AE33 manufacturer default C = 1.39 (green dashed line). Grey shading indicates periods when the daily mean k ≤ 0 resulting from the high aerosol single scattering albedo, during which C cannot be reliably determined following Ferrero et al. (2024). **(c)** Daily median eBC derived from the AE33 factory settings (C = 1.39, MAC = 7.77 $m^2\ g^{-1}$; grey line) and after correction (C = 2.952, MAC = 8.8 $m^2\ g^{-1}$; blue line).

**S7 Overview of the land observation station and the ship cruise track in the Arctic.**

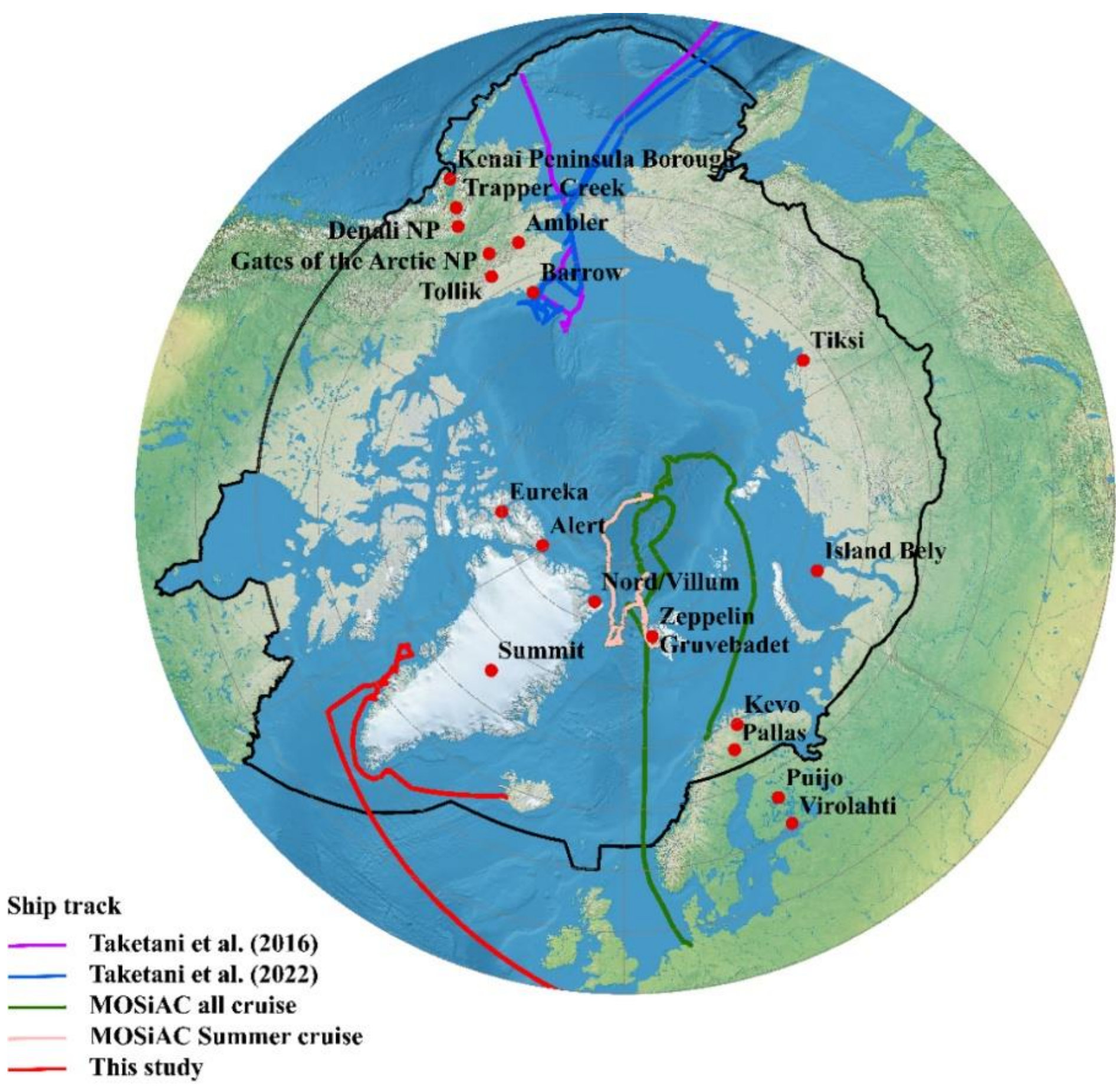


**Fig. S18** Overview of the land observation station and the ship cruise track in the Arctic. The black boundary is the definition of the Arctic region from the Arctic Monitoring and Assessment Program (AMAP) (https://www.amap.no/work-area/document/868, last access: 7 July 2025). The base map is from Natural Earth II (https://www.naturalearthdata.com/, last access: 30 June 2025).

**Table S3** Overview of BC mass concentrations based on land-site observations in the Arctic. Here we mainly focused on observations during summer.

| Location | latitude | longitude | BC (ng/m$^{-3}$) | Measure period | Instrument | Method | Inlet | Data sources |
|---|---|---|---|---|---|---|---|---|
| Utö | 59.78° N | 21.38° E | 187±161 | Summer 2008 | AE31 | Station | $PM_{2.5}$ | Hyvärinen et al. (2011) |
| Kenai Peninsula Borough | 60.01° N | 151.71°W | 96.8 | May-July, 2022 | Filter analyzed by Model 2015 multiwavelength carbon analyzer | Station | $PM_{2.5}$ | IMPROVED aerosol |
| Virolahti | 60.52° N | 27.68° E | 297±292 | Summer 2008 | AE31 | Station | $PM_{2.5}$ | Hyvärinen et al. (2011) |
| Hyytiälä | 61.85° N | 24.29° E | 219±187 | Summer 2008 | AE31 | Station | $PM_{10}$ | Hyvärinen et al. (2011) |
| Trapper Creek | 62.32° N | 150.32°W | 147.6 | May-July, 2022 | Filter analyzed by Model 2015 multiwavelength carbon analyzer | Station | $PM_{2.5}$ | IMPROVED aerosol |
| Puijo | 62.90° N | 27.65° E | 174±154 | Summer 2008 | MAAP, λ=370 nm | Station | $PM_{2.5}$ | Hyvärinen et al. (2011) |
| Denali NP | 63.72°N | 148.97°W | 93.5 | May-July, 1988-1999 | Filter analyzed by Sunset OC–EC | Station | $PM_{2.5}$ | IMPROVED aerosol |
| | | | 111.6 | May-July, 2000-2015 | | | | |
| | | | 112.2 | May-July, 2016-2019 | Filter analyzed by Model 2015 multiwavelength carbon analyzer | | | |
| | | | 263.9 | May-July, 2022 | | | | |
| | | | 97.8 | May-July, 2020-2024 | | | | |
| Gates of the Arctic NP | 66.9°N | 151.52°W | 111.0 | May-July, 2009-2015 | Filter analyzed by Sunset OC–EC | Station | $PM_{2.5}$ | IMPROVED aerosol |
| Pallas | 67.97°N | 24.12°E | 3.3 | 2008-2009 | MAAP | Station | / | Eckhardt et al. (2015) |
| | | | 26 | Winter 2011-2012 | SP2 | Station | $PM_{10}$ | Raatikainen et al. (2015) |
| | | | 60 ± 65 | Summer 2008 | AE31 | Station | Total | Hyvärinen et al. (2011) |

**Table S3** Continued.

| Location | latitude | longitude | BC (ng/m$^{-3}$) | Measure period | Instrument | Method | Inlet | Data sources |
|---|---|---|---|---|---|---|---|---|
| Pallas | 67.97°N | 24.12°E | 61 | July-August, 2006-2010 | Whatman 42 Filters analyzed by Optical Transmissometer, OT21 | Station | / | Dutkiewicz et al. (2014) |
| Abisko | 68.36°N | 19.05°E | 19 ± 3.6 | 12 June – 28 August, 2012 | Sunset standard thermal−optical transmission (TOT) analyzer | Station | $PM_{2.5}$ | Winiger et al. (2016) |
| Ambler | 68.63°N | 149.61°W | 531.5 | July 2004 | Filter analyzed by Sunset OC–EC | Station | $PM_{2.5}$ | FED IMPROVED aerosol |
| Toolik Lake Field Station | 68.63°N | 149.61°W | 570.9 | May-July, 2022 | Filter analyzed by Model 2015 multiwavelength carbon analyzer | Station | $PM_{2.5}$ | FED IMPROVED aerosol |
| Kevo | 69.76°N | 27.01°E | 127 | Summer, 1964 to 2010 | Whatman 42 Filters analyzed by Optical Transmissometer, OT21 | Station | / | Dutkiewicz et al. (2014) |
| Barrow | 71.32°N | 156.61°W | 5.5 | June-July 2009 | PSAP | Station | / | Eckhardt et al. (2015) |
| | | | 32.7 | May, 2010-2014 | AE31 | Station | / | Xu et al. (2017) |
| | | | 16.8 | May 2009-2015 | PSAP | | | |
| | | | 33.0 | June, 2010-2014 | AE31 | | | |
| | | | 10.5 | June 2009-2015 | PSAP | | | |

**Table S3** Continued.

| Location | latitude | longitude | BC (ng/m$^{-3}$) | Measure period | Instrument | Method | Inlet | Data sources |
|---|---|---|---|---|---|---|---|---|
| Barrow | 71.32°N | 156.61°W | 9.3 ± 12.0 | Summer, 2012-2015 | COSMOS | Station | $PM_1$ | Sinha et al. (2017) |
| | | | <2 | June-July, 2020 | SP2 | Station | / | Sedlacek et al. (2021) |
| Tiksi | 71.36°N | 128.53°E | 15.3 | 31 May – 2 June, 2015 | Aethalometer | Station | TSP | Popovicheva et al. (2019) |
| | | | 50 | 2009-2018; 2015-2020 | AE31, λ=880nm | Station | $PM_{10}$ | Schmale et al. (2022) |
| | | | 37.8 | 21 June 2012 | Sunset standard thermal−optical transmission (TOT) analyzer | Station | TSP | Winiger et al. (2017) |
| | | | 20-40 | Summer 2009-2010 | Aethalometer | Station | / | Eckhardt et al. (2015) |
| Summit | 72.58°N | 38.46°W | 50 | 2012-2020; 2011-2020 | CLAP, λ=550nm | Station | $PM_{2.5}$ | Schmale et al. (2022) |
| Island Bely | 73.33 °N | 70.67 °E | 30 | July, 2020 | AE33 | Station | / | Schacht et al. (2019) |
| Zeppelin (475 m)/ Ny-Ålesund (10m) | 78.90 °N | 11.88 °E | 6.9 | June-July 2009 | PSAP | Station | / | Eckhardt et al. (2015) |
| | | | 7.0 ± 3.5 | Summer 2017-2020 | Sunset OC/EC | Station | $PM_{10}$ | Yttri et al. (2024) |
| | | | 21.9 | May, 2009-2010 | AE31 | Station | / | Xu et al. (2017) |
| | | | 15.4 | May 2009-2014 | PSAP | | | |
| | | | 14.7 | June, 2009-2010 | AE31 | | | |
| | | | 8.6 | June 2009-2014 | PSAP | | | |
| | | | 6.2 ± 7.9 | Summer, 2012-2015 | COSMOS | Station | $PM_1$ | Sinha et al. (2017) |
| Eureka | 80.0°N | 85.9°W | 30.1 | April | SP2 | Aircraft | / | Schulz et al. (2019) |

**Table S3** Continued.

| Location | latitude | longitude | BC (ng/m$^{-3}$) | Measure period | Instrument | Method | Inlet | Data sources |
|---|---|---|---|---|---|---|---|---|
| Nord (Villum) | 81.6°N | 16.67°W | 27.4 | June-July 2009 | Weekly filter analyzed by Sunset OC–EC | Station | / | Sinha et al. (2017) |
| | | | 19.8 | March 2008- February 2010 | PSAP | Station | / | Nguyen et al. (2013) |
| | | | 20 | May 2015 | SP2 | Station | / | Nielsen et al. (2019) |
| | | | 20 ± 22 | June, July, August, 2011-2013 | Weekly filter analyzed by Sunset OC–EC | Station | $PM_{10}$ | Massling et al. (2015) |
| | | | 11 ± 9 | | MAAP, λ=670 nm | Station | TSP | |
| Alert | 82.5°N | 62.5°W | 7.5 | June-September, 1989-2002 | AE06 | Station | / | Sharma (2004) |
| | | | 13 | Summer 2011-2013 | AE31 | Station | TSP | Sharma et al. (2017) |
| | | | 6 | | SP2 | Station | $PM_1$ | |
| | | | 19 | | OC-EC analyser | Station | $PM_1$ | |
| | | | 33.7 | May, 2011-2013 | AE31 | Station | / | Xu et al. (2017) |
| | | | 27.9 | May, 2011-2013 | Thermal_EC* | | $PM_1$ | |
| | | | 12.1 | May 2011-2013 | SP2 | | / | |
| | | | 19.0 | May, 2011-2013 | PSAP | | / | |
| | | | 14.3 | June, 2011-2013 | AE31 | | / | |
| | | | 21.0 | June, 2011-2013 | Thermal_EC* | | $PM_1$ | |
| | | | 6.0 | June 2011-2013 | SP2 | | / | |
| | | | 9.5 | June, 2011-2013 | PSAP | | / | |
| | | | 4 | June-July 2009 | PSAP | Station | / | Eckhardt et al. (2015) |

**Table S4** Overview of BC mass concentrations based on shipborne observations in the Arctic. Here we mainly focused on observations during summer.

| Region | Period | BC | Instrument | Referecence |
|---|---|---|---|---|
| Western Arctic Ocean (70–75° N, ~170–156° W) | September 2014 | 1.0 ± 1.2 | SP2 | Taketani et al. (2016) |
| Western Arctic Ocean (70–75° N, ~170–153° W) | September 2015 | 0.9 ± 1.4 | SP2 | Taketani et al. (2022) |
| Western Arctic Ocean (70–74° N, ~170–152° W) | September 2016 | 0.7 ± 1.8 | SP2 | Taketani et al. (2022) |
| Central Arctic | June 2020 | 24.79 ± 23.46 | Aethalometer | MOSAiC campaign |
| Russian High Arctic | 9–25 October 2015 | <30 | Aethalometer | Popovicheva et al. (2017) |
| the Arctic Ocean (< 80° N and 166° E to 156° W) | summer and early autumn of 2016–2020 | > 70 | COSMOS and Aethalometer | Deng et al. (2024) |
| Western Arctic Ocean (82.88°N,180–136°W) | 25 July–31 August 2016 | 23 ± 55 | Aethalometer | Ding et al. (2018) |

## S8 representativeness of Greenland emission

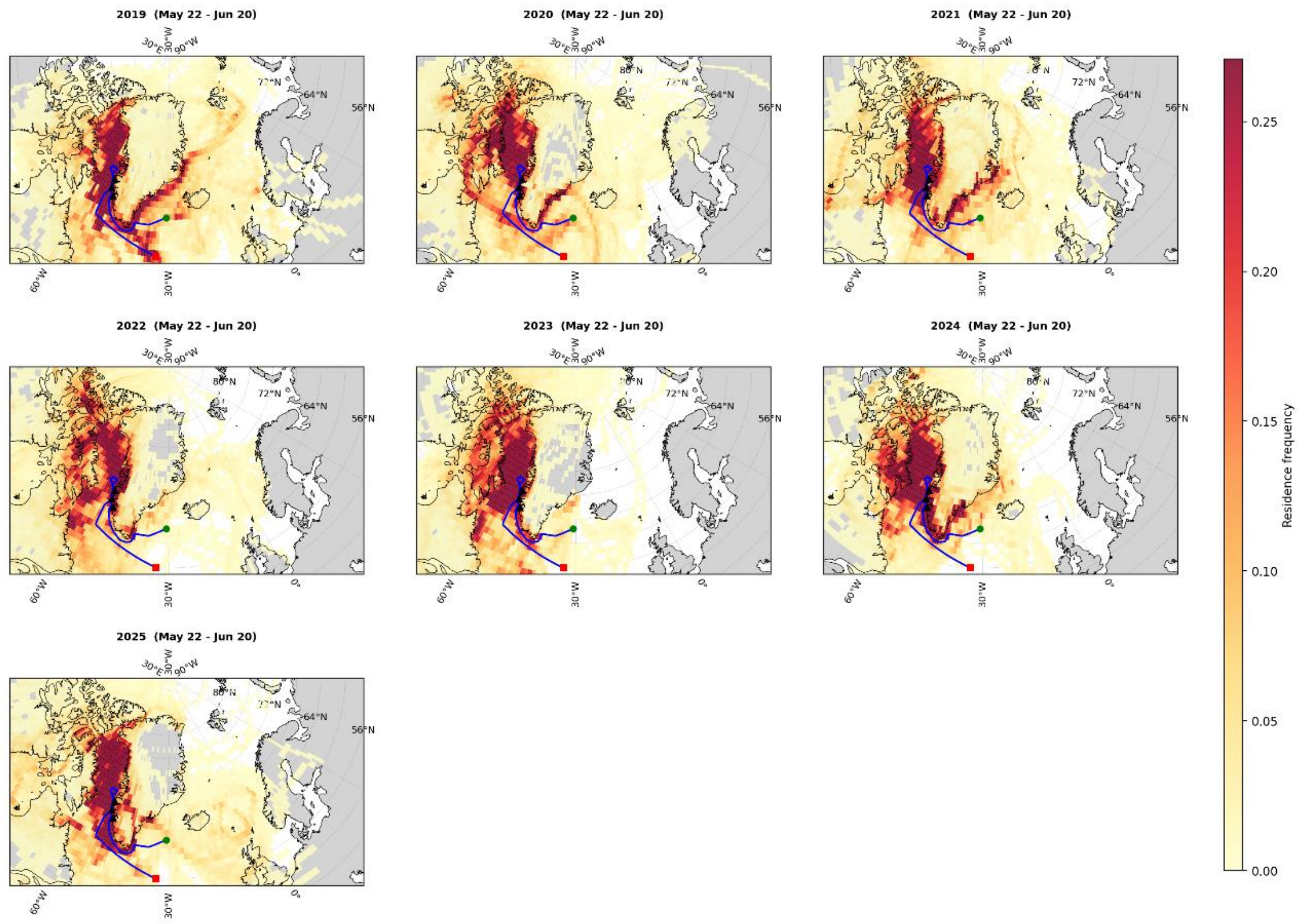


**Fig. S19** Ten-day backward trajectory residence time frequency for the same cruise track (May 22–June 20) across 2019–2025. Trajectories were initialized at 10 m release height at each hourly ship position using HYSPLIT with GDAS meteorological data. Color shading indicates the fraction of trajectories passing through each 1°×1° grid cell.

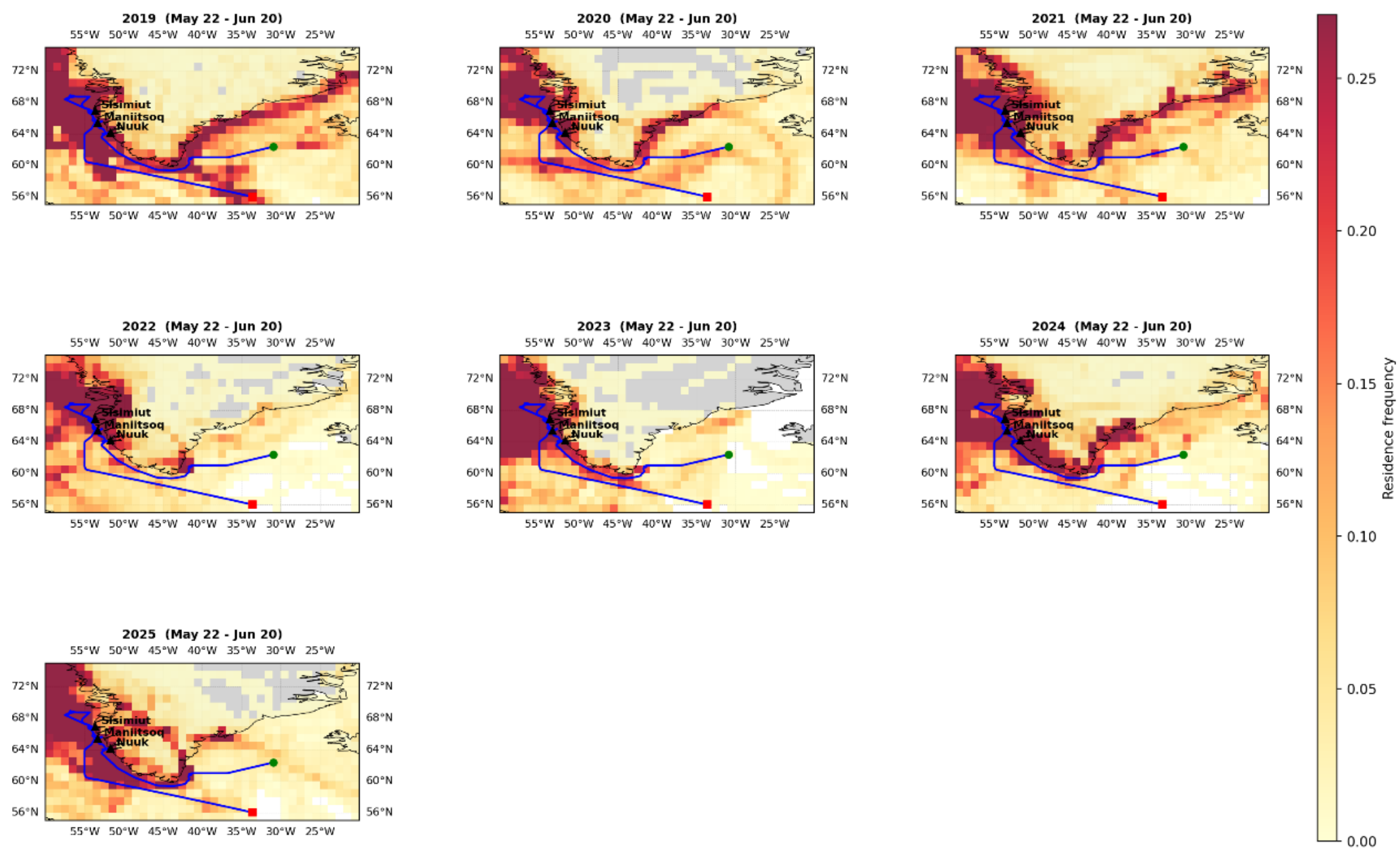

Fig. S20 As Figure F19, zoomed in to the Greenland region.

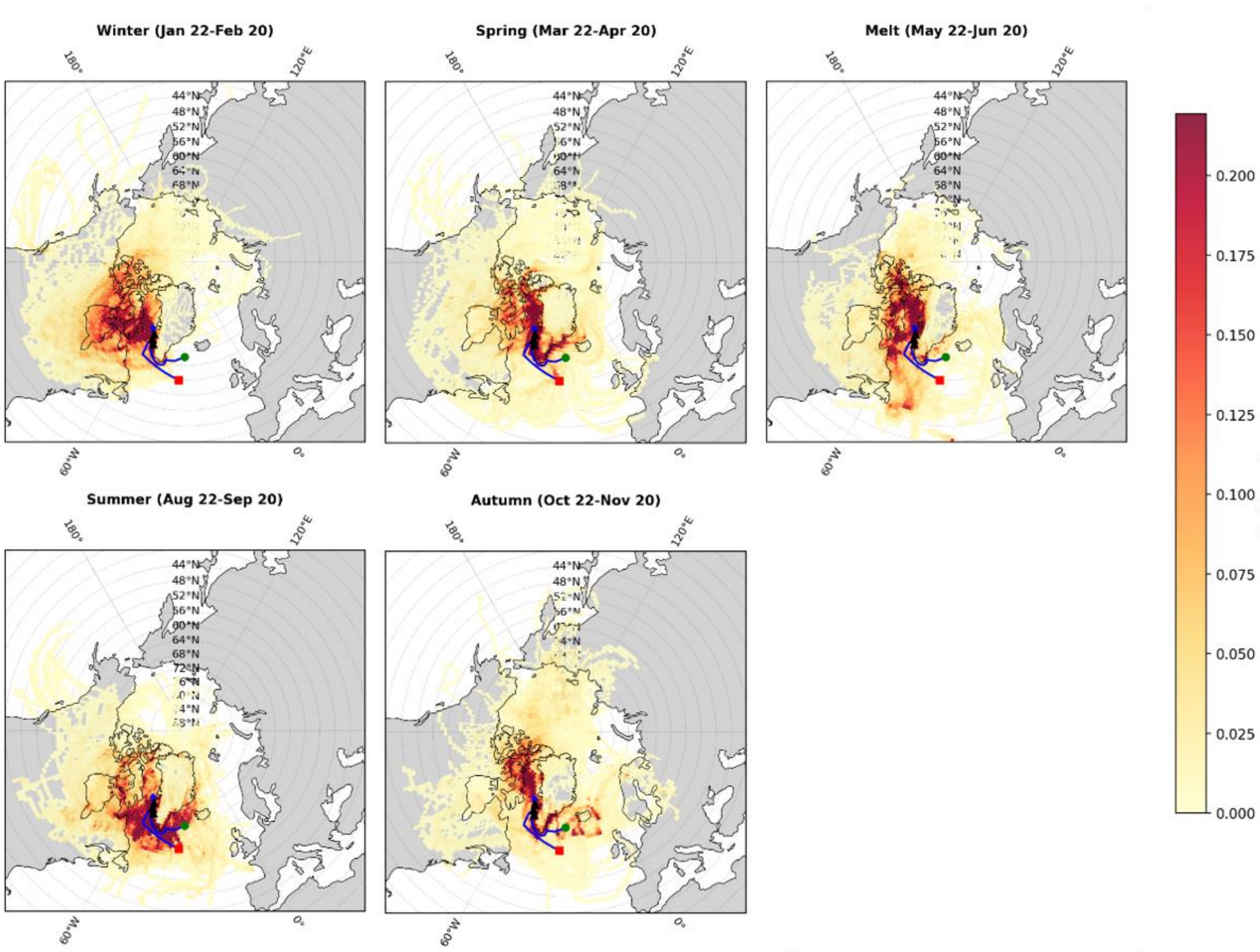


**Fig. S21** Ten-day backward trajectory residence time frequency for the same cruise track shifted to five seasons in 2022: winter (Jan 22–Feb 20), spring (Mar 22–Apr 20), melting season (May 22–Jun 20), summer (Aug 22–Sep 20), and autumn (Oct 22–Nov 20). Trajectory initialization and analysis methods are as described in Figure S19.

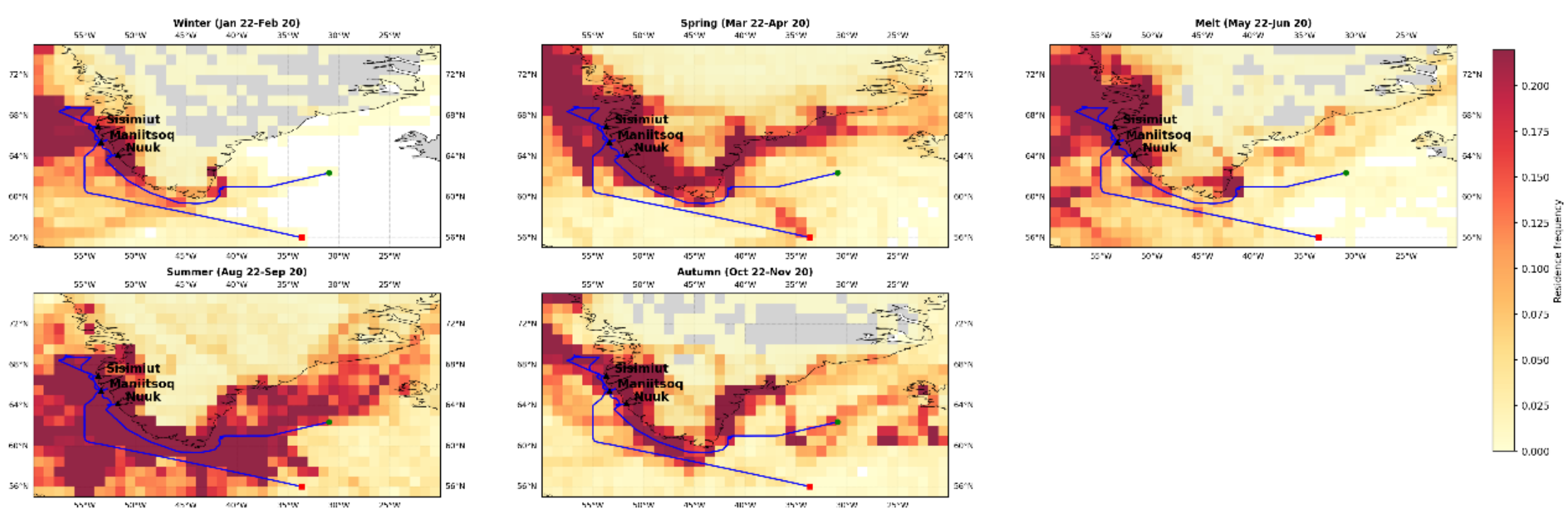


**Fig. S22** As Figure S21, zoomed in to the Greenland region.

## Reference

Deng, Y., Tanimoto, H., Ikeda, K., Kameyama, S., Okamoto, S., Jung, J., Yoon, Y. J., Yang, E. J., & Kang, S.-H. (2024). Shipborne observations of black carbon aerosols in the western Arctic Ocean during summer and autumn 2016–2020: Impact of boreal fires. *Atmospheric Chemistry and Physics*, *24*(10), 6339–6357. https://doi.org/10.5194/acp-24-6339-2024

Ding, M., Tian, B., Zhang, T., Tang, J., Peng, H., Bian, L., & Sun, W. (2018). Shipborne observations of atmospheric black carbon aerosol from Shanghai to the Arctic Ocean during the 7th Chinese Arctic Research Expedition. *Atmospheric Research*, *210*, 34–40. https://doi.org/10.1016/j.atmosres.2018.04.016

Dutkiewicz, V. A., DeJulio, A. M., Ahmed, T., Laing, J., Hopke, P. K., Skeie, R. B., Viisanen, Y., Paatero, J., & Husain, L. (2014). Forty-seven years of weekly atmospheric black carbon measurements in the Finnish Arctic: Decrease in black carbon with declining emissions. *Journal of Geophysical Research: Atmospheres*, *119*(12), 7667–7683. https://doi.org/10.1002/2014JD021790

Eckhardt, S., Quennehen, B., Olivié, D. J. L., Berntsen, T. K., Cherian, R., Christensen, J. H., Collins, W., Crepinsek, S., Daskalakis, N., Flanner, M., Herber, A., Heyes, C., Hodnebrog, Ø., Huang, L., Kanakidou, M., Klimont, Z., Langner, J., Law, K. S., Lund, M. T., … Stohl, A. (2015). Current model capabilities for simulating black carbon and sulfate concentrations in the Arctic atmosphere: A multi-model evaluation using a comprehensive measurement data set. *Atmospheric Chemistry and Physics*, *15*(16), 9413–9433. https://doi.org/10.5194/acp-15-9413-2015

Hyvärinen, A.-P., Kolmonen, P., Kerminen, V.-M., Virkkula, A., Leskinen, A., Komppula, M., Hatakka, J., Burkhart, J., Stohl, A., Aalto, P., Kulmala, M., Lehtinen, K. E. J., Viisanen, Y., & Lihavainen, H. (2011). Aerosol black carbon at five background measurement sites over Finland, a gateway to the Arctic. *Atmospheric Environment*, *45*(24), 4042–4050. https://doi.org/10.1016/j.atmosenv.2011.04.026

IMPROVE (Interagency Monitoring of Protected Visual Environments). IMPROVE Aerosol Network Dataset; Cooperative Association for Studies of the Atmosphere (CIRA), Colorado State University: Fort Collins, CO, 2024. https://vista.cira.colostate.edu/Improve/ (accessed 2024-07-01).

Massling, A., Nielsen, I. E., Kristensen, D., Christensen, J. H., Sørensen, L. L., Jensen, B., Nguyen, Q. T., Nøjgaard, J. K., Glasius, M., & Skov, H. (2015). Atmospheric black carbon and sulfate concentrations in Northeast Greenland. *Atmospheric Chemistry and Physics*, *15*(16), 9681–9692. https://doi.org/10.5194/acp-15-9681-2015

Nguyen, Q. T., Skov, H., Sørensen, L. L., Jensen, B. J., Grube, A. G., Massling, A., Glasius, M., & Nøjgaard, J. K. (2013). Source apportionment of particles at Station Nord, North East Greenland during 2008–2010 using COPREM and PMF analysis. *Atmospheric Chemistry and Physics*, *13*(1), 35–49. https://doi.org/10.5194/acp-13-35-2013

Nielsen, I. E., Skov, H., Massling, A., Eriksson, A. C., Dall'Osto, M., Junninen, H., Sarnela, N., Lange, R., Collier, S., Zhang, Q., Cappa, C. D., & Nøjgaard, J. K. (2019). Biogenic and

anthropogenic sources of aerosols at the High Arctic site Villum Research Station. *Atmospheric Chemistry and Physics*, *19*(15), 10239–10256. https://doi.org/10.5194/acp-19-10239-2019

Popovicheva, O. B., Evangeliou, N., Eleftheriadis, K., Kalogridis, A. C., Sitnikov, N., Eckhardt, S., & Stohl, A. (2017). Black Carbon Sources Constrained by Observations in the Russian High Arctic. *Environmental Science & Technology*, *51*(7), 3871–3879. https://doi.org/10.1021/acs.est.6b05832

Popovicheva, O., Diapouli, E., Makshtas, A., Shonija, N., Manousakas, M., Saraga, D., Uttal, T., & Eleftheriadis, K. (2019). East Siberian Arctic background and black carbon polluted aerosols at HMO Tiksi. *Science of The Total Environment*, *655*, 924–938. https://doi.org/10.1016/j.scitotenv.2018.11.165

Raatikainen, T., Brus, D., Hyvärinen, A.-P., Svensson, J., Asmi, E., & Lihavainen, H. (2015). Black carbon concentrations and mixing state in the Finnish Arctic. *Atmospheric Chemistry and Physics*, *15*(17), 10057–10070. https://doi.org/10.5194/acp-15-10057-2015

Schacht, J., Heinold, B., Quaas, J., Backman, J., Cherian, R., Ehrlich, A., Herber, A., Huang, W. T. K., Kondo, Y., Massling, A., Sinha, P. R., Weinzierl, B., Zanatta, M., & Tegen, I. (2019). The importance of the representation of air pollution emissions for the modeled distribution and radiative effects of black carbon in the Arctic. *Atmospheric Chemistry and Physics*, *19*(17), 11159–11183. https://doi.org/10.5194/acp-19-11159-2019

Schulz, H., Zanatta, M., Bozem, H., Leaitch, W. R., Herber, A. B., Burkart, J., Willis, M. D., Kunkel, D., Hoor, P. M., Abbatt, J. P. D., & Gerdes, R. (2019). High Arctic aircraft measurements characterising black carbon vertical variability in spring and summer. *Atmospheric Chemistry and Physics*, *19*(4), 2361–2384. https://doi.org/10.5194/acp-19-2361-2019

Sedlacek, A. J. (2021). *North Slope of Alaska Black Carbon Loadings and Mixing State for MOSAiC Field Campaign Report* (DOE/SC-ARM-21-014). Oak Ridge National Laboratory (ORNL), Oak Ridge, TN (United States). ARM Data Center. https://doi.org/10.2172/1810321

Sharma, S. (2004). Long-term trends of the black carbon concentrations in the Canadian Arctic. *Journal of Geophysical Research*, *109*(D15), D15203. https://doi.org/10.1029/2003JD004331

Sharma, S., Leaitch, W. R., Huang, L., Veber, D., Kolonjari, F., Zhang, W., Hanna, S. J., Bertram, A. K., & Ogren, J. A. (2017). An evaluation of three methods for measuring black carbon in Alert, Canada. *Atmospheric Chemistry and Physics*, *17*(24), 15225–15243. https://doi.org/10.5194/acp-17-15225-2017

Sinha, P. R., Kondo, Y., Koike, M., Ogren, J. A., Jefferson, A., Barrett, T. E., Sheesley, R. J., Ohata, S., Moteki, N., Coe, H., Liu, D., Irwin, M., Tunved, P., Quinn, P. K., & Zhao, Y. (2017). Evaluation of ground-based black carbon measurements by filter-based photometers at two Arctic sites. *Journal of Geophysical Research: Atmospheres*, *122*(6), 3544–3572. https://doi.org/10.1002/2016JD025843

Taketani, F., Miyakawa, T., Takashima, H., Komazaki, Y., Pan, X., Kanaya, Y., & Inoue, J. (2016).

Shipborne observations of atmospheric black carbon aerosol particles over the Arctic Ocean, Bering Sea, and North Pacific Ocean during September 2014: BLACK CARBON OVER ARCTIC OCEAN. *Journal of Geophysical Research: Atmospheres*, *121*(4), 1914–1921. https://doi.org/10.1002/2015JD023648

Taketani, F., Miyakawa, T., Takigawa, M., Yamaguchi, M., Komazaki, Y., Mordovskoi, P., Takashima, H., Zhu, C., Nishino, S., Tohjima, Y., & Kanaya, Y. (2022). Characteristics of atmospheric black carbon and other aerosol particles over the Arctic Ocean in early autumn 2016: Influence from biomass burning as assessed with observed microphysical properties and model simulations. *Science of The Total Environment*, *848*, 157671. https://doi.org/10.1016/j.scitotenv.2022.157671

Winiger, P., Andersson, A., Eckhardt, S., Stohl, A., & Gustafsson, Ö. (2016). The sources of atmospheric black carbon at a European gateway to the Arctic. *Nature Communications*, *7*(1), 12776. https://doi.org/10.1038/ncomms12776

Winiger, P., Andersson, A., Eckhardt, S., Stohl, A., Semiletov, I. P., Dudarev, O. V., Charkin, A., Shakhova, N., Klimont, Z., Heyes, C., & Gustafsson, Ö. (2017). Siberian Arctic black carbon sources constrained by model and observation. *Proceedings of the National Academy of Sciences*, *114*(7). https://doi.org/10.1073/pnas.1613401114

Xu, J.-W., Martin, R. V., Morrow, A., Sharma, S., Huang, L., Leaitch, W. R., Burkart, J., Schulz, H., Zanatta, M., Willis, M. D., Henze, D. K., Lee, C. J., Herber, A. B., & Abbatt, J. P. D. (2017). Source attribution of Arctic black carbon constrained by aircraft and surface measurements. *Atmospheric Chemistry and Physics*, *17*(19), 11971–11989. https://doi.org/10.5194/acp-17-11971-2017

Yttri, K. E., Bäcklund, A., Conen, F., Eckhardt, S., Evangeliou, N., Fiebig, M., Kasper-Giebl, A., Gold, A., Gundersen, H., Myhre, C. L., Platt, S. M., Simpson, D., Surratt, J. D., Szidat, S., Rauber, M., Tørseth, K., Ytre-Eide, M. A., Zhang, Z., & Aas, W. (2024). Composition and sources of carbonaceous aerosol in the European Arctic at Zeppelin Observatory, Svalbard (2017 to 2020). *Atmospheric Chemistry and Physics*, *24*(4), 2731–2758. https://doi.org/10.5194/acp-24-2731-2024

Yus-Díez, J., Bernardoni, V., Močnik, G., Alastuey, A., Ciniglia, D., Ivančič, M., Querol, X., Perez, N., Reche, C., Rigler, M., Vecchi, R., Valentini, S., & Pandolfi, M. (2021). Determination of the multiple-scattering correction factor and its cross-sensitivity to scattering and wavelength dependence for different AE33 Aethalometer filter tapes: A multi-instrumental approach. *Atmospheric Measurement Techniques*, *14*(10), 6335–6355. https://doi.org/10.5194/amt-14-6335-2021
